# Trivalent and quadrivalent influenza vaccination effectiveness in Australia and South Africa: results from a modelling study

**DOI:** 10.1111/irv.12367

**Published:** 2016-02-08

**Authors:** George J. Milne, Nilimesh Halder, Joel K. Kelso, Ian G. Barr, Jocelyn Moyes, Kathleen Kahn, Rhian Twine, Cheryl Cohen

**Affiliations:** ^1^ School of Computer Science and Software EngineeringUniversity of Western AustraliaPerthWAAustralia; ^2^ World Health Organization (WHO) Collaborating Centre for Reference and Research on InfluenzaMelbourneVic.Australia; ^3^ Centre for Respiratory Disease and MeningitisNational Institute for Communicable DiseasesJohannesburgSouth Africa; ^4^ MRC/Wits Rural Public Health and Health Transitions Research Unit (Agincourt)Faculty of Health ScienceSchool of Public HealthUniversity of the WitwatersrandJohannesburgSouth Africa; ^5^ Faculty of Health ScienceSchool of Public HealthUniversity of the WitwatersrandJohannesburgSouth Africa

**Keywords:** Influenza vaccination, quadrivalent influenza vaccine, seasonal influenza

## Abstract

**Background:**

A modelling study was conducted to determine the effectiveness of trivalent (TIV) and quadrivalent (QIV) vaccination in South Africa and Australia.

**Objectives:**

This study aimed to determine the potential benefits of alternative vaccination strategies which may depend on community‐specific demographic and health characteristics.

**Methods:**

Two influenza A and two influenza B strains were simulated using individual‐based simulation models representing specific communities in South Africa and Australia over 11 years. Scenarios using TIV or QIV, with alternative prioritisation strategies and vaccine coverage levels, were evaluated using a country‐specific health outcomes process.

**Results:**

In South Africa, approximately 18% fewer deaths and hospitalisations would be expected to result from the use of QIV compared to TIV over the 11 modelled years (*P* = 0·031). In Australia, only 2% (*P* = 0·30) fewer deaths and hospitalisations would result. Vaccinating 2%, 5%, 15% or 20% of the population with TIV using a strategy of prioritising vulnerable age groups, including HIV‐positive individuals, resulted in reductions in hospitalisations and mortality of at least 7%, 18%, 57% and 66%, respectively, in both communities.

**Conclusions:**

The degree to which QIV can reduce health burden compared to TIV is strongly dependent on the number of years in which the influenza B lineage in the TIV matches the circulating B lineages. Assuming a moderate level of B cross‐strain protection, TIV may be as effective as QIV. The choice of vaccination prioritisation has a greater impact than the QIV/TIV choice, with strategies targeting those most responsible for transmission being most effective.

## Introduction

Seasonal influenza is an infectious respiratory illness responsible for an estimated 250 000–500 000 deaths globally each year.^1^ Young children, the elderly and those with other underlying health conditions such as HIV have an increased risk of developing complications of influenza, such as pneumonia.^2^, ^3^, ^4^

Vaccination to mitigate seasonal influenza is widely used in some countries, with 20% or more of the population being vaccinated annually in Australia, the UK and the United States, for example.^5^, ^6^, ^7^ The most common vaccine in use over the last 30 years has been a trivalent inactivated vaccine (TIV) containing three vaccine strains: two influenza A strains [e.g. A(H1N1) and A(H3N2)] and an influenza B strain. The specific viral strain and lineage for each subtype are updated on an bi‐annual cycle (for the Northern and Southern Hemispheres) based on currently circulating strains.^8^ Since 2001, two distinct influenza B lineages (Yamagata and Victoria) have cocirculated, with one or both lineages causing a significant proportion of influenza infection in each year.^9^, ^10^, ^11^, ^12^, ^13^, ^14^, ^15^ In years when the predominant B lineage is incorrectly predicted, or where it is correctly predicted but there is a significant proportion of the other lineage, the TIV for that year leaves vaccinated individuals vulnerable to influenza infection from the ‘missing’ B lineage, since cross‐protection between B lineages is limited.^16^ To address this issue, quadrivalent influenza vaccines (QIV), which include two influenza A and two influenza B strains, have been developed and are currently available.^11^, ^12^

Many countries are considering the introduction or expansion of seasonal influenza vaccination programmes (e.g. South Africa, where annual coverage is approximately 2%^17^), or the replacement of TIV with QIV. The potential for seasonal influenza vaccination to mitigate the health burden of influenza depends on factors such as vaccine efficacy and the proportion of the population vaccinated each year, its coverage. It may also depend on specific community characteristics such as demographics, household size, health care infrastructure and the prevalence of comorbid health conditions that predispose individuals to severe complications in the event of influenza infection. The potential benefits of QIV versus TIV have not previously been examined in Australia or South Africa, for have QIV and TIV vaccination strategies been compared between countries.

## Methods

In this study, we modelled seasonal influenza spread and influenza vaccination in two communities: the low‐income rural community of Agincourt, South Africa, where influenza vaccination coverage is low (<2%); and the town of Albany, Western Australia, a community in a high‐income country where TIV is currently used (with overall coverage approximately 20% ranging from <5% for children to >75% for the elderly) and where QIV is being considered for future use. Two individual‐based, dynamic influenza transmission models, which include multiple circulating influenza strains calibrated using data from each of the two countries, are used to simulate influenza transmission and predict the age‐specific incidence of symptomatic influenza infection.

For each community, we consider a range of vaccination scenarios, representing choices that face public health authorities. These are the choice of vaccine, TIV or QIV; vaccination coverage levels, ranging stepwise from 2% to 20% of the population; and the choice of vaccination prioritisation strategy, that is the order in which different population groups are prioritised for vaccination when there is a fixed, and perhaps limited, supply of vaccine.

**Figure 1:**
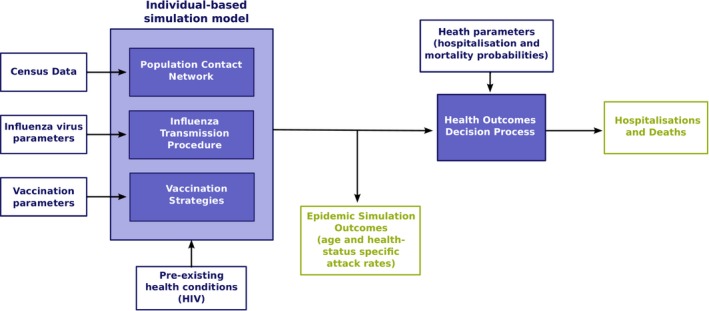
Overview of study methodology.

For each vaccination scenario, a community‐specific health outcomes process is used to estimate the potential reduction in the health burden of seasonal influenza in terms of symptomatic cases, hospitalisations and death, due to use of either TIV or QIV, as in Figure 1. Full details of the study methodology are given in Supporting information.

## Results

### Reduction in health burden due to vaccination

Table 1 shows the estimated symptomatic infection, hospitalisation and mortality rates due to influenza infection, in each community for the TIV and QIV vaccination scenarios, averaged over the 11 years (2003–2013). This TIV strategy reduces symptomatic influenza infection by 49% (from 4·9% to 2·5%) in South Africa and by 47% (from 4·9% to 2·6%) in Australia, a result of significance to South Africa given the limited influenza vaccination occurring there.

**Table 1:** Symptomatic attack rates for 15% vaccination coverage using HIV/vulnerable‐first prioritisation strategy

Vaccination scenario	Attack rate	Hospitalisation rate	Mortality rate
	% of population (95% CI)	Per 100 000 (% reduction from NV)	Per 100 000 (% reduction from NV)
Agincourt, South Africa			
NV (no vaccination)	4·9 (4·7–5·1)	60·4	26·1
TIV	2·5 (2·3–2·7)	25·8 (57)	11·2 (57)
QIV	2·2 (1·9–2·4)	21·0 (65)	9·2 (65)
Albany, Australia			
NV (no vaccination)	4·9 (4·6–5·2)	215·5	11·6
TIV	2·6 (2·3–2·9)	91·5 (57)	4·7 (59)
QIV	2·5^a^ (2·3–2·8)	89·5^a^ (58)	4·6^a^ (60)

Values are symptomatic attack rate (as % of the population, with 95% confidence interval for variation due to simulation stochasticity over 40 simulation runs), hospitalisation rate (per 100 000) and mortality rate (per 100 000) attributable to influenza averaged over 11 years. Vaccination coverage is 15%.

a The outcome for quadrivalent influenza vaccine (QIV) is not statistically significantly different from that of trivalent inactivated vaccine (TIV) due to stochastic simulation variation (*P* > 0·3).

The hospitalisation and mortality rates vary between the two communities due to differences in demographics, community health characteristics and availability of hospital resources for severe influenza cases; see the Discussion section for further explanation. However, the *relative reduction* in serious health outcomes is similar between the communities, with TIV more than halving hospitalisation and mortality rates.

### Quadrivalent influenza vaccine versus trivalent inactivated vaccine

From Table 1, it can be seen that the additional reduction in cases (i.e. the symptomatic attack rate) and health burden due to use of QIV compared to TIV are larger in South Africa compared to Australia. In South Africa, vaccinating 15% of the population with QIV using a strategy of prioritising HIV‐positive and elderly individuals is expected to result in 12%, 18·6% and 17·8% fewer illnesses, hospitalisations and deaths, respectively, compared to TIV (*P* < 0·05, null hypothesis that QIV is not more effective than TIV and that apparent advantage of QIV is due to stochastic simulation variation). In Australia, the same QIV vaccination scenario yields a smaller 3·8%, 2·2% and 2·1% reduction in illness, hospitalisation and death, respectively, compared to TIV; these smaller additional reductions in health burden are not highly significant (*P* > 0·3). Reasons for these differences are detailed in the Discussion section.

**Figure 2:**
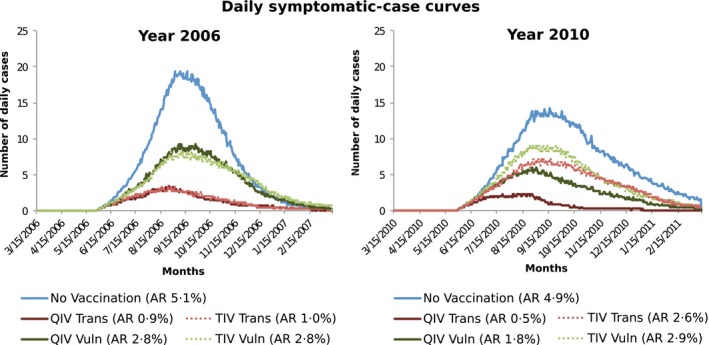
Epidemic curves for years with trivalent inactivated vaccine (TIV) influenza B lineage match and mismatch.

Although the results shown in Table 1 are averaged over 11 years, the advantage of QIV over TIV in any particular year depends strongly on which B lineages circulated for a given year *and* the particular B lineage included in that year's TIV. Figure 2 illustrates this phenomenon using epidemic curves from the simulation of the influenza seasons in 2006 and 2010 in the South African community.

In 2006, the dominant strain was A(H3N2), with small amounts of A(H1N1) and B Victoria, and negligible B Yamagata. The TIV included the B Victoria strain in that year; as a consequence, the trivalent vaccine performed as well as the quadrivalent one, as seen in the left panel of Figure 2 by the dotted epidemic curves representing TIV overlying the solid curves representing QIV.

In 2010, all four strains circulated in approximately equal proportions. As the trivalent vaccine included the B Victoria strain, the circulating B Yamagata strain was not covered by the trivalent vaccine. In the right panel of Figure 2, the dotted TIV epidemic curves lie above the solid QIV curves, the difference representing infections caused by the (missing from TIV) B Yamagata lineage. Simulation outcome attack rate data for each of the 11 years are presented in Tables S5 and S6.

The Australian and South African models differ on both the ratio of influenza strains circulating each year and the characteristics of each community. To determine which of these factors were determining the effectiveness of QIV versus TIV, we repeated the main analysis using South African strain ratios in the Australian model and Australian strain ratios in the South African model. The results (summarised in Table S9) show that when the same strain ratios are used in both communities, the benefit of TIV versus QIV was similar.

### Vaccination coverage and prioritisation

Table 2 shows estimated influenza attack rates for the HIV/vulnerable‐first and transmitters‐first prioritisation strategies and vaccination coverage of 2%, 5%, 15% and 20%, for both models.

**Table 2:** Health outcomes for alternative TIV vaccination coverage and prioritisation strategies

Vaccination scenario	Transmitters‐first			HIV/vulnerable‐first		
	AR	HR	MR	AR	HR	MR
Agincourt, South Africa						
NV	4·9	60·4	26·1	4·9	60·4	26·1
2%	4·0	50·1	21·2	4·2	51·1	22·2
5%	2·8	36·1	14·9	3·6	41·7	18·5
15%	1·4	18·8	7·7	2·5	25·8	11·2
20%	1·2	15·2	6·7	1·9	19·9	8·5
Albany, Australia						
None	4·9	215·5	11·6	4·9	215·5	11·6
2%	3·9	174·0	9·4	4·7	200·2	10·7
5%	2·2	99·4	5·4	4·5	175·7	9·1
15%	0·7	33·1	1·8	2·6	91·5	4·7
20%	0·6	28·6	1·6	2·0	72·6	3·8

NV, no vaccination.

Result values are estimated symptomatic attack rate (AR, as % of the population) and hospitalisation rate (HR, per 100 000) and mortality rate (MR, per 100 000) attributable to influenza averaged over 11 years. The vaccination strategy is trivalent inactivated vaccine (TIV) with 15% coverage.

For both communities and all vaccination coverage levels, the transmitters‐first vaccination strategy was more effective in minimising the attack rate compared to the HIV/vulnerable‐first strategy. It was also more effective than the other two strategies simulated (vulnerable‐first and random vaccine prioritisation, see Tables S2–S4). The transmitters‐first strategy also resulted in the largest reduction in hospitalisation and death, with the exception that in South Africa, at 2% vaccination coverage, preferentially vaccinating with the transmitters‐first strategy, is not significantly different from the HIV/vulnerable‐first strategy, resulting in 50·1 compared to 51·1 hospitalisations per 100 000 population, respectively.

### Cross‐protection between B lineages

In prior scenarios, no B lineage cross‐strain vaccine protection was assumed, that is vaccination with TIV did not provide any protection against the influenza B strain not included in the vaccine, and vaccine efficacy for that strain was zero. Recent studies^18^, ^19^ suggest that TIVs offer cross‐protection against non‐vaccine B lineages. McLean *et al*.^18^ found 51% vaccine effectiveness against the non‐vaccine B lineage for the 2012/2013 influenza season in the United States and Skowronski *et al*.^19^ found 27% vaccine effectiveness against the non‐vaccine B lineage for the 2011/2012 influenza season in Canada. Given these results, we conducted sensitivity analyses assuming TIV offers 7%, 13%, 26% or 52% effectiveness against non‐vaccine B lineages. The outcomes, given in Table 3, suggest that with a modest level of cross‐protection, TIV can offer the same level of attack rate reduction achieved by QIV. For example, if 26% vaccine effectiveness against the non‐vaccine B lineages is maintained, the attack rates for TIV are no more than 0·1% of the population higher than QIV, for every scenario.

**Table 3:** Symptomatic attack rates with varying degree of TIV B lineage cross‐protection

Priority Strategy	NV	TIV, cross‐protection against non‐TIV B lineage			QIV
		0% VE	26% VE	52% VE	
Agincourt, South Africa					
Trans	4·9 (4·7–5·1)	1·4 (1·3–1·6)	1·0^a^ (0·8–1·1)	0·9^a^ (0·8–1·0)	0·9 (0·8–1·1)
H‐Vuln	4·9 (4·7–5·1)	2·5 (2·3–2·7)	2·1^a^ (1·9–2·3)	2·1^a^ (1·9–2·4)	2·2 (1·9–2·4)
Albany, Australia					
Trans	4·9 (4·6–5·2)	0·7 (0·6–0·8)	0·6^a^ (0·5–0·7)	0·6^a^ (0·5–0·7)	0·6 (0·5–0·7)
H‐Vuln	4·9 (4·6–5·2)	2·7 (2·4–2·9)	2·6^a^ (2·3–2·9)	2·6^a^ (2·5–2·9)	2·6 (2·4–2·9)

NV, no vaccination; TIV, trivalent influenza vaccine; QIV, quadrivalent influenza vaccine; Trans, transmitters‐first; H‐Vuln, HIV/vulnerable‐first.

Result values are estimated symptomatic attack rate as % of the population (with 95% confidence interval) attributable to influenza averaged over 11 years. Vaccination coverage is 15%.

a TIV attack rates with 26% or higher cross‐protection VE against the non‐TIV influenza B lineages are not statistically significantly (95% confidence level) different from QIV attack within the limits of stochastic simulation variability.

### Sensitivity to influenza and vaccination parameters

The impact of the alternative assumptions examined is summarised in Table 4. The finding that the transmitters‐first vaccination prioritisation strategy provided the greatest reduction in influenza illness and health burden was found to be consistent across all sensitivity analysis settings. Further explanation of sensitivity analysis simulation results is given in Supporting information.

**Table 4:** Alternative parameter settings

Sensitivity analysis scenario	Effect on vaccination effectiveness	Effect on QIV versus TIV relative effectiveness
Cross‐protection against non‐TIV B lineage	TIV more effective	TIV as effective as QIV if cross‐protection VE of at least 26%
Higher no‐vaccination attack rate	Larger absolute attack rate reduction; lower relative attack rate reduction	None
Lower vaccine efficacy	Vaccination less effective	None
Waning immunity	Vaccination less effective	None
Pre‐existing immunity lower in younger population	Transmitters‐first prioritisation more effective; vulnerable‐first prioritisation less effectives	None

QIV, quadrivalent influenza vaccine; TIV, trivalent inactivated vaccine.

## Discussion

### Key findings

In South Africa, setting the study suggested significant reductions in deaths and hospitalisations resulting from the use of QIV compared to TIV over the 11 modelled years. Vaccinating 15% of the population with QIV using a strategy of prioritising HIV‐positive and elderly individuals is expected to result in 12%, 18·6% and 17·8% fewer illnesses, hospitalisations and deaths, respectively, compared to TIV. In Australia, the same QIV vaccination scenario gives a smaller benefit of 3·8%, 2·2% and 2·1% reduction in illness, hospitalisation and death, respectively, compared to TIV.

The reason for this difference is that the B lineage component of the TIV, which was the same in both communities and based on the WHO Southern Hemisphere recommendations,^8^ was less well matched to the circulating South African B strains than the Australian strains for the 11 years of the study. There were more mismatched years in South Africa in which either (i) there was significant cocirculation of both B strains or (ii) only one B strain circulated, but the TIV B component was for the other B strain (as show in Supporting information, Table S3). In these mismatched years, TIV was less effective, and there was greater scope for QIV to compensate for the B strain missing from the vaccine. In South Africa, in seven of the 11 study years, the percentage of circulating influenza B that was not included in the TIV was greater than 5%, while in Australia, this was only 3 of the 11 years. These results indicate that the value of QIV over TIV is crucially dependent on which B lineages circulate in the season following vaccination.

The fact that the differences in QIV advantage are determined by the strain ratios and not demographic differences in the respective community models is confirmed by the fact that when Australian strain ratios were used, the difference between QIV and TIV was 0·1% of the population in both community models; when South African ratios were used, the difference was 0·5% and 0·4% in the Australian and South African community models, respectively.

However, if some degree of cross‐strain protection exists between the B lineage contained in the TIV and the other circulating lineage, then the benefit of QIV over TIV is less significant, with cross‐strain efficacy of at least 26% TIV found to be almost as effective as QIV over the 11‐year period considered, in both South Africa and Australia.

A strategy of vaccinating 15% of the population with TIV using a HIV/vulnerable‐first strategy results in a reduction in hospitalisation and mortality of at least 57% in both communities (see Table 1). Although the percentage by which the number of hospitalisation and deaths is reduced compared to the no‐vaccination level is similar in both communities, the *absolute* reduction varies between communities. Due to older population and higher case hospitalisation, the absolute reduction in hospitalisations in Australia was estimated to be 124 per 100 000 (215·5 down to 91·5) compared to 34·6 per 100 000 in South Africa (60·4 down to 25·8). In contrast, higher influenza case mortality rates (particularly in HIV‐positive individuals) in South Africa result in larger absolute reductions in mortality in South Africa (from 26·1 down to 11·2 per 100 000) compared to Australia (11·6 down to 4·7 per 100 000). Further discussion on hospitalisation and mortality rates in each model is given in Supporting information.

This study has examined the effectiveness of influenza vaccination in the Southern Hemisphere, where such data are limited. In South Africa, a middle‐income country with a high prevalence of HIV and very low rates of influenza vaccination, we have considered a low‐income rural community, the first such study. The model of a low‐income rural community in South Africa, with a *per capita* income of less than $1000 per annum, is likely also reflective of other countries in sub‐Saharan Africa. The study is also the first to examine the relative effectiveness of QIV versus TIV in Australia, an industrialised country with high levels of seasonal influenza vaccination.

### Vaccination prioritisation

For both communities, for both TIV and QIV, and for vaccination coverage levels of 5% and above, the greatest reduction in influenza burden is achieved by the transmitters‐first prioritisation strategy (see Tables 2 and S4–S6). While a greater number of vulnerable individuals are directly protected from infection with the vulnerable‐first prioritisation strategies, the larger attack rate reduction achieved by prioritising those age classes most responsible for transmission results in more hospitalisations and deaths prevented. The greater effectiveness of the transmitters‐first prioritisation strategy is due to the indirect herd immunity effect, which is only captured by dynamic transmission models (see Supporting information for further discussion of the impact of herd immunity effects and vaccination prioritisation). The potential benefits of vaccinating children are increasingly being recognised and some countries, for example the UK,^20^ are adopting school‐aged child vaccination programmes. Our model suggests that this could be considered for other settings, as the benefits are significant with even modest levels of vaccination coverage.

### Related research

Reed *et al*.^21^ estimated the benefits of TIV and QIV using coverage, vaccine efficacy and health outcomes data from the USA. As a static model was used, no indirect herd immunity effects were modelled, and the benefit of vaccination with both TIV and QIV was found to be smaller than those presented here. Their study estimated that QIV prevented 0·04 deaths per 100 000 population per year compared to TIV. The scenario most comparable in our study is the Albany model with 15% TIV using a vulnerable‐first priority, which gave a reduction of 0·1 deaths per 100 000 per year (see Table S6). This value is larger than that found by Reed *et al*. but not significantly so given stochastic simulation variation in our model.

Van Bellinghen *et al*.^22^ and Meier *et al*.^23^ are related studies using the same static, multicohort Markov model based on UK data, and also assumed no herd immunity effects. They included a detailed model of population age demographics and comorbid health conditions which followed the modelled population year by year for a lifetime (up to 100 years). This study estimated that QIV prevented an additional 0·26 deaths per 100 000 population per year compared to TIV: our value of 0·1 deaths per 100 000 is smaller but not significantly so.

Eichner *et al*.^24^, ^25^ used an individual‐based dynamic transmission model somewhat similar to ours to estimate the effectiveness of TIV versus QIV in Germany, but ran the model over a significant time period, allowing for new strain emergence year by year. This study estimated that the use of QIV versus TIV would prevent an additional 0·49% (reducing the TIV attack rate of 2·6–2·1%). The frequency of B lineage mismatch in the model was high (50% chance of vaccine mismatch each year) as was vaccination coverage (25% growing to 30% over the simulation period); the closest matching scenario in our study was the South African model with 20% vaccination coverage and a vulnerable‐first strategy. This gave a QIV versus TIV attack rate advantage of 0·4%, which is not significantly different from the value estimated by Eicher *et al*.

### Limitations and future work

While we used actual influenza strain ratio and vaccine composition data for the modelled period, each of the simulated years was otherwise treated the same. In particular, the demographics, community health characteristics and seasonal influenza attack rate were assumed to be constant. We believe our analysis is appropriate at the current time (2015) when the introduction QIV and/or expansion of TIV is being considered. Changes in future decades due to ageing demographics or improved community health would be a cause for re‐analysis.

The use of a constant 5% symptomatic attack rate for all years and the alternative 10% presented in Supporting Information are simplifying assumptions, since the scale of seasonal influenza varies from year to year and from country to country. The best estimates available^6^, ^9^ are based on surveys that correlate serological RNA RT‐PCR testing and prospective community surveillance. However, these studies cover at most 5 years and we have chosen to apply average values from these studies to all 11 years considered in our study. Horby *et al*.^9^ estimated an average annual infection rate of 21·5% and a symptomatic attack rate of 3·17% over 3 years (2007, 2008 and 2010); this study gives an estimate in a setting (Vietnam) where no seasonal influenza vaccination was used. Hayward *et al*.^6^ estimated an annual infection rate 18%, with 25% of illnesses being asymptomatic, averaged over 5 years (2006–2011). The fact that a approximately 4·5% symptomatic attack rate was observed in a setting with TIV (the UK, with 20% coverage) motivated us to consider an alternative no‐vaccination attack rate of 10%.

We have adopted a simple model of immunity carrying over between influenza seasons, with individuals having a 27% chance of being immune to each strain at the start of each year (see Supporting information section ‘Influenza assumptions used in the study’). It is possible that this underestimates the amount of immunity present in population. With more immunity existing in the community each season, the transmissibility (i.e. *R*_0_) of circulating strains would have to be higher in order to give the assumed 5% or 10% attack rates. This might potentially impact on our estimates of vaccine effectiveness, since the higher transmissibility would render vaccination somewhat less effective. It seems unlikely, however, that this effect would impact the relative effectiveness of TIV versus QIV. If either or both B lineages had higher rates of pre‐existing immunity than influenza A, this would impact both TIV and QIV.

Another phenomenon that might result in increased immunity in the population is viral interference, which is a short‐term non‐specific innate immune response to viral infection. Influenza viral interference is hypothesised to explain patterns of seasonal and pandemic incidence and strain replacement,^26^, ^27^ and observations of increased incidence of other respiratory infections associated with seasonal influenza vaccination.^28^ The simulation model used in the current study includes viral interference, as infection with one strain excludes infection by others for the duration of infection. However, if viral interference occurs for a more extended duration, it may contribute to higher overall levels of immunity and thus imply higher levels of influenza transmissibility and possible lower vaccine efficacy via the mechanism outlined in the previous paragraph.

One phenomenon that could potentially affect the relative effectiveness of TIV versus QIV is the existence of *cross‐protection specifically between B lineages* due to infection. That is, if infection with one B lineage offers a degree of protection against the other. We are not aware of any field studies that estimate influenza B lineage cross‐protection; however, a recent study has demonstrated an *in vitro* mechanism by which it can occur.^29^ In a year in which substantial amounts of both B lineages are circulating, each may be partially holding the other in check through cross‐protection. A TIV would reduce incidence of one lineage, but this might then cause a ‘rebound’ of the other due to reduced cross‐protection. This effect would blunt the impact of the TIV, but not QIV.

We have used HIV as the only comorbidity as it occurs with high prevalence in South Africa, and we have high‐quality data indicating poor outcomes for HIV‐positive individuals following influenza infection. Given limited vaccine supplies, we found that vaccination targeting the HIV cohort was highly effective in reducing the case, hospitalisation and death rate. We also considered vaccination strategies that targeted age‐specific risk groups, namely the elderly, children under 5 years of age and those infected with HIV. There are, however, other important vulnerable groups, such as pregnant women and conferring maternal immunity to infants under 6 months, and those with other comorbid health conditions including the following: chronic pulmonary obstructive disease, diabetes, asthma and malnutrition. Future research will consider this wider range of comorbidities.

Our results on TIV effectiveness may be applicable in countries with similar demographic and health systems: the characteristics of the South African rural Agincourt model are similar to many settings in sub‐Saharan Africa, while the Australian model is similar to other industrialised countries. In generalising the results of QIV versus TIV, the local circulation of influenza B lineages *relative to the composition of the TIV vaccine used in the community* is probably more important than community characteristics. Our results based on Australian strain circulation and TIV composition may be applicable for settings with good TIV B lineage matches, while the South African results may be more applicable for settings with significant mismatches.

## Conclusions

Due to the unpredictable nature of influenza strain circulation, the benefit of a QIV over a trivalent vaccine cannot be predicted ahead of time. We have shown that the benefit of QIV over TIV is strongly related to which B lineages circulate in a given year and the lineage contained in the TIV. In South Africa, where seven of 11 years had a substantial B lineage mismatch, vaccinating 15% of the population with QIV using a strategy of prioritising HIV‐positive and elderly individuals may be expected to result in 18% fewer hospitalisations and deaths compared to TIV. In Australia, where only 3 years had significant TIV B lineage mismatches, the same QIV vaccination scenario yields only a 2% reduction in hospitalisation and death, compared to TIV.

An important finding of this study is that if the influenza B lineage included in the TIV provides even a low level of cross‐protection against the other B lineage, then TIV *may* perform as well as QIV.

This study indicates that the current Australian practice of vaccinating 20% of its population with a trivalent influenza vaccine has reduced hospitalisation and death attributable to seasonal influenza *by at least half*. The benefit of significantly increasing TIV use in South Africa, and countries with similarly high levels of comorbidities such as HIV, is likely to be even greater in terms of reduction in hospitalisation and death per vaccine dose.

When comparing alternative vaccination strategies, the herd immunity benefit of vaccination targeted at children, who contribute most to onward transmission, is significant. Our results suggest that in both communities and for coverage levels of 5% and above, a transmitters‐first TIV strategy is preferable to QIV using a vulnerable‐first prioritisation, resulting in fewer hospitalisations and deaths.

## Conflict of interests

The authors declare that they have no conflict of interests.

## Author contributions

GJM and CC conceived and designed the study. GJM, JKK and NH were responsible for the design of the simulation experiments and analysis of the results. CC, JM, KK and RT provided community model and health data. NH was responsible for software development and conducted the simulation experiments. JKK wrote the initial draft of the manuscript. All authors read, contributed to and approved the final manuscript.

## Supporting information


**Data S1.** Detailed methodology, additional results and sensitivity analyses.
**Table S1.** Model parameters and main analysis scenario values.
**Table S2.** Age‐specific health parameters.
**Table S3.** ‐ Percentage of influenza B lineages and linage used in TIV.
**Table S4.** Estimated influenza attack rates by community and vaccination scenario.
**Table S5.** Estimated respiratory infection hospitalisations due to influenza by community and TIV vaccination scenario.
**Table S6.** Estimated mortality due to influenza by community and vaccination scenario.
**Table S7.** Attack rates for Agincourt, transmitters‐first, coverage 15%.
**Table S8.** Attack rates for Albany, transmitters‐first, coverage 15%.
**Table S9.** Advantage of QIV versus TIV for combinations of community model and influenza circulation.
**Table S10.** Age breakdown of hospitalisation and death per 100 000 population in Agincourt for 15% vaccination coverage.
**Table S11.** Age breakdown of hospitalisation and death per 100 000 population in Albany for 15% vaccination coverage.
**Table S12.** Hospitalisation per 100 000 population in Agincourt and Albany for 15% vaccination coverage and for different levels of cross protection.
**Table S13.** Deaths per 100 000 population in Agincourt and Albany for 15% vaccination coverage and for different levels of cross protection.
**Table S14.** Hospitalisation per 100 000 population in Agincourt and Albany for 15% vaccination coverage and for lower vaccine effectiveness (i.e. 51% for <65 age groups and 26% for 65+ age groups).
**Table S15.** Deaths per 100 000 population in Agincourt and Albany for 15% vaccination coverage and for lower vaccine effectiveness (i.e. 51% for <65 age groups and 26% for 65+ age groups).
**Table S16.** Hospitalisation per 100 000 population in Agincourt and Albany for 15% vaccination coverage and for subtype specific vaccine effectiveness.
**Table S17.** Deaths per 100 000 population in Agincourt and Albany for 15% vaccination coverage and for subtype specific vaccine effectiveness.
**Table S18.** Hospitalisation per 100 000 population in Agincourt and Albany for 40% vaccination coverage.
**Table S19.** Deaths per 100 000 population in Agincourt and Albany for 40% vaccination coverage.
**Table S20.** Hospitalisation per 100 000 population in Agincourt and Albany for 15% vaccination coverage and 10% symptomatic attack rate.
**Table S21.** Deaths per 100 000 population in Agincourt and Albany for 15% vaccination coverage and 10% symptomatic attack rate.
**Table S22.** Hospitalisation per 100 000 population in Agincourt and Albany for 15% vaccination coverage and waning immunity scenario.
**Table S23.** Deaths per 100 000 population in Agincourt and Albany for 15% vaccination coverage and waning immunity scenario.
**Table S24.** Hospitalisation per 100 000 population in Agincourt and Albany for alternative Pre‐existing immunity assumption.
**Table S25.** Deaths per 100 000 population in Agincourt and Albany for alternative Pre‐existing immunity assumption.
