# Supplementary material for: Trivalent and quadrivalent influenza vaccination effectiveness in Australia and South Africa: results from a modelling study
Source: Influenza Other Respir Viruses. 2016 Feb 8;10(4):324–32. doi: 10.1111/irv.12367 (PMC4910176; doi:10.1111/irv.12367)
Supplement: Supplementary file 1 — Data S1. Detailed methodology, additional results and sensitivity analyses. Table S1. Model parameters and main analysis scenario values. Table S2. Age‐specific health parameters. Table S3. ‐ Percentage of influenza B lineages and linage used in TIV. Table S4. Estimated influenza attack rates by community and vaccination scenario. Table S5. Estimated respiratory infection hospitalisations due to influenza by community and TIV vaccination scenario. Table S6. Estimated mortality due to influenza by community and vaccination scenario. Table S7. Attack rates for Agincourt, transmitters‐first, coverage 15%. Table S8. Attack rates for Albany, transmitters‐first, coverage 15%. Table S9. Advantage of QIV versus TIV for combinations of community model and influenza circulation. Table S10. Age breakdown of hospitalisation and death per 100 000 population in Agincourt for 15% vaccination coverage. Table S11. Age breakdown of hospitalisation and death per 100 000 population in Albany for 15% vaccination coverage. Table S12. Hospitalisation per 100 000 population in Agincourt and Albany for 15% vaccination coverage and for different levels of cross protection. Table S13. Deaths per 100 000 population in Agincourt and Albany for 15% vaccination coverage and for different levels of cross protection. Table S14. Hospitalisation per 100 000 population in Agincourt and Albany for 15% vaccination coverage and for lower vaccine effectiveness (i.e. 51% for <65 age groups and 26% for 65+ age groups). Table S15. Deaths per 100 000 population in Agincourt and Albany for 15% vaccination coverage and for lower vaccine effectiveness (i.e. 51% for <65 age groups and 26% for 65+ age groups). Table S16. Hospitalisation per 100 000 population in Agincourt and Albany for 15% vaccination coverage and for subtype specific vaccine effectiveness. Table S17. Deaths per 100 000 population in Agincourt and Albany for 15% vaccination coverage and for subtype specific vaccine effectiveness. Table S18. Hospi [file IRV-10-324-s001.docx]

Trivalent and quadrivalent influenza vaccination effectiveness in Australia and South Africa: results from a modelling study

Detailed Methology 1

Community Models 1

Multi-strain seasonal influenza transmission 3

Influenza assumptions used in the study 5

Vaccine assumptions 7

Vaccination prioritisation 8

Health outcomes decision process 9

Simulation experiments 9

Additional Results 10

Sensitivity Analyses 15

Vaccination B-lineage cross protection: Tables S12 and S13 15

Vaccine efficacy: Tables S14 to S17 15

Higher 40% vaccination coverage: Tables S18 and S19 15

Alternative symptomatic attack rate: Tables S20 and S21 15

Waning immunity: Tables S22 and S23 15

Pre-existing immunity: Tables S24 and S25 16

Additional Discussion 22

Herd immunity effects 22

Hospitalisation and Mortality Rates 22

HIV/vulnerable first strategy 23

References 24

# Detailed Methology

## Community Models

Each community model replicates an actual community with census and other data used to capture the age, occupation and health status of each individual, and their membership of one or more contact groups. These consist of people who are in regular daily contact such as in a specific household, school class, or workplace. These virtual community models were developed to match the real communities on which they are based as closely as possible, using country-specific census and other available data.

We used an individual-based, multi-strain influenza spread simulation mechanism to simulate the spread of influenza and the effect of influenza vaccination in each community model. Each model captures the infection history of each individual i.e. if infected, and by what subtype; when infected; where infected; and their infection status each day, either susceptible, infected but not yet infectious (the latent period), infectious, or immune (due to infection or vaccination). The output of the influenza spread model includes a day-by-day count of the number of influenza infections and the number of resulting cases (i.e. symptomatic influenza infections), categorised by the age and health status of the individual. Based on this simulation output, an age-specific health outcomes process for each community was used to estimate the health burden due to influenza infection in terms of hospitalisation and death. Key model parameters are listed in Tables S1 and S2.

We ran an ensemble of simulation experiments using both models, with each experiment representing a different vaccination scenario. These examined the type of vaccine (TIV vs. QIV), the vaccination coverage (the percentage of the population vaccinated), and how vaccine was prioritised among different groups in the community. We have used the best available data from studies of seasonal influenza and vaccination to select plausible model parameter values. For parameters that are likely to impact strongly on the effectiveness of vaccination effectiveness we have performed a series of sensitivity analysis simulation experiments which examined alternative, plausible parameter value settings.

A model represents each individual in a community and captures the demographics, household structure, and the locales in which individuals come into contact with others and potentially transmit influenza. The influenza spread simulation system used in this study has been used previously in pandemic influenza models of the towns of Albany in Western Australia ^1^ and Madang in Papua New Guinea (PNG) ^2^. Comparison of these two models has shown that community characteristics such as household size and the population’s age structure strongly influence influenza spread and the effectiveness of potential influenza mitigation measures ^2^. Differences in population age structure and the prevalence of co-morbidities (that increase the risk of poor outcomes following influenza infection) mean that the impact of seasonal influenza and the effectiveness of influenza vaccination need to be evaluated using models that represent distinct characteristics of specific countries. We thus expect results of this study to be broadly applicable to both developing countries (e.g. sub-Saharan Africa) and developed countries. It should be noted that while we simulate seasonal influenza transmission in the period 2003-2013, we assume that the demographics of the modelled communities are static, being based on 2004 census data in the case of the Australian model and 2008 survey data in the South African model (see below).

Each community is modelled using *data structures* which capture detailed demographic information for each individual and the structure of households, schools and workplaces, such as the age of each individual, in one of 7 age classes (0-5, 6-12, 13- 17, 18- 24, 25-44, 45-64, 65+); whether the individual has been vaccinated; resulting immunity to A and B strains due to vaccination or infection; which individuals make up each household; the location of each household; which individuals meet daily in each school class or workplace; and the health status of each individual, i.e. the presence of co-morbidities such as HIV or underlying lung disease ^3, 4^. A *simulation* *algorithm* computes the time-varying infection history of each individual, and this is used to determine the daily number of cases occurring in the overall community, giving an epidemic curve, as in Figure 2. Details specific to each of the two models are as follows:

***Agincourt***

The Agincourt community model represents a collection of villages in the Bushbuckridge rural municipality in the north of South Africa. This community is representative of low-income rural populations in South Africa, and elsewhere in sub-Saharan Africa. The selected community contains 11 villages with a total population of 40,383 and includes pre-schools, schools and health clinics. The villages selected are part of the Agincourt South African Demographic Surveillance Site (DSS). As a consequence, high quality data on the demographics, health, education, and major employers in the community was available from surveys conducted from 2008 to 2012 ^5, 6^. Census data for 2011 from Statistics South Africa was also used ^7^.

To represent the day-time location and interpersonal contacts between unemployed adults and children not attending school (comprising 56% of the adult and 23% of the school-aged population respectively), additional neighbourhood hubs were added to the school and workplace hubs in the model, as used in our previous PNG model ^2^. The most significant co-morbidity in South Africa predisposing individuals to serious influenza complications is HIV (~16% of the adult population); age-specific prevalence data for HIV was used to assign a co-morbidity health status to each individual ^4, 8^.

***Albany***

The Albany, Australia community model has been described in previous publications, for example in ^1, 9-13^. As with the South African model, age specific HIV prevalence data for Australia was used to assign co-morbidity status to individuals ^14, 15^.

## Multi-strain seasonal influenza transmission

A simulation algorithm computes changes to the underlying data structures capturing population mobility and the time-changing infectivity profile of each individual. Thus the algorithm models the dynamics of influenza transmission within the community. Each individual has their infectivity status denoted by one of four states (susceptible, exposed, infectious, recovered/immune) at two time points 12 hours apart, for each day of the simulated period. Separate infectivity status was recorded *for each of the 4 influenza strains*. The simulation algorithm captures the location and infection status of the whole population twice per day, a daytime point-in-time snapshot and an evening snapshot, with individuals (possibly) moving locations between successive day or night periods, such as household to school or workplace for the day phase, returning home for the night period. Individuals come into contact with other individuals on a one-to-one basis in each location, with *possible* influenza transmission then occurring. Individuals occupying the same location during the same time period are assumed to come into potential infective contact, and influenza may be transmitted from infectious to susceptible individuals according to a probability function which characterises the inherent transmissibility of that strain ^1, 10^.

This individual-based influenza spread modelling methodology has been used previously in a series of studies examining many aspects of pandemic influenza spread and mitigation, including vaccination e.g. ^10, 12, 13^. In this study the models have additional features: the co-circulation of 4 influenza strains; the effect of multi-valent vaccines; and use of an individual’s health information, e.g. HIV infection status.

Multi-strain influenza transmission and vaccination modelling methods are described below. Additional simulation model details are presented in e.g. ^1^. Assumed model parameters are as follows:

**Table S1 - Model parameters and main analysis scenario values**

| **Parameters** | **Values** | **Source** |
| --- | --- | --- |
| infection rate | ~21% | ^16, 17^ |
| symptomatic attack rate | ~5% | ^16, 17^ |
|  |  |  |
| **Epidemiological parameters** |  |  |
| Symptomatic infectiousness timeline | 0.5 day latent (non-infectious), 1 day asymptomatic; 2 days peak | ^18^ |
| Asymptomatic infectiousness timeline | 0.5 day latent; 5.5 days asymptomatic | ^18^ |
| asymptomatic infectiousness | 0.5 | ^18^ |
| peak symptomatic infectiousness | 1.0 | - |
| post-peak symptomatic infectiousness | 0.5 | ^18^ |
| probability of asymptomatic infection | 0.77 | ^16, 17^ |
| probability withdrawal if symptomatic | 0.5 for adults; 0.9 for child | - |
| proportion of population immune at start of season (for each strain) | 27% | ^19, 20^ |
| **Interpersonal contact parameters** |  |  |
| school class mixing group size | 10 | ^1^ |
| maximum workplace mixing group size | 10 | ^1^ |
| community contacts per person per day | 4 | ^1^ |
| **Vaccination parameters** |  |  |
| vaccine efficacy | 65% for <65 age groups; 55% for 65+ age groups | ^21-23^ |
| vaccination coverage | 15% (1%, 2%, 5%, 20%) | - |

**Table S2 Age-Specific health parameters**

| **Parameters** | **Values** | | **Source** |
| --- | --- | --- | --- |
| **Prevalence of HIV infection** | **Agincourt** | **Albany** |  |
| Age (0-5) | 4.43% | 0.0038% | Agincourt  ^24^  Albany  ^14, 15^ |
| Age (6-12) | 5.28% | 0.0042% |  |
| Age (13-17) | 5.28% | 0.1206% |  |
| Age (18-24) | 5.28% | 0.3315% |  |
| Age (25-44) | 26.21% | 0.2399% |  |
| Age (45-64) | 12.96% | 0.1040% |  |
| Age 65+ | 1.42% | 0.0253% |  |
| **Symptomatic ase hospitalisation probability (HIV-)** | **Agincourt** | **Albany** |  |
| Age (0-5) | 0.044519 | 0.043066 | Agincourt  ^8^  Albany  ^25^ |
| Age (6-12) | 0.002091 | 0.003733 |  |
| Age (13-17) | 0.001552 | 0.005777 |  |
| Age (18-24) | 0.001898 | 0.0088 |  |
| Age (25-44) | 0.002578 | 0.00914 |  |
| Age (45-64) | 0.010081 | 0.027997 |  |
| Age 65+ | 0.013511 | 0.493768 |  |
| **Symptomatic case hospitalisation probability (HIV+)** | **Agincourt** | **Albany** |  |
| Age (0-5) | 0.09943 | 0.1572 |  |
| Age (6-12) | 0.02329 | 0.0438 |  |
| Age (13-17) | 0.01687 | 0.06787 |  |
| Age (18-24) | 0.02121 | 0.1036 |  |
| Age (25-44) | 0.04254 | 0.29628 |  |
| Age (45-64) | 0.06569 | 0.19712 |  |
| Age 65+ | 0.10136 | - |  |
| **Symptomatic case fatality probability (HIV-)** | **Agincourt** | **Albany** |  |
| Age (0-5) | 0.001258 | 0.000482 | Agincourt^1^  ^3^  Albany^2^  ^26, 27^ |
| Age (6-12) | 0.000098 | 0.000042 |  |
| Age (13-17) | 0.000073 | 0.000062 |  |
| Age (18-24) | 0.000114 | 0.000084 |  |
| Age (25-44) | 0.000146 | 0.000091 |  |
| Age (45-64) | 0.008281 | 0.000478 |  |
| Age 65+ | 0.104855 | 0.035629 |  |
| **Symptomatic case fatality probability (HIV+)** | **Agincourt** | **Albany** |  |
| Age (0-5) | 0.018338 | 0.008363 |  |
| Age (6-12) | 0.004659 | 0.001945 |  |
| Age (13-17) | 0.003373 | 0.002898 |  |
| Age (18-24) | 0.008442 | 0.005512 |  |
| Age (25-44) | 0.011837 | 0.011956 |  |
| Age (45-64) | 0.046472 | 0.002682 |  |
| Age 65+ | 0 | - |  |

^1^ Additional data on influenza mortality in ages 5 years+ supplied by the National Institute of Communicable Diseases, South Africa.

^2^ Relative risks of hospitalisation and death for HIV infected individuals were not available for Australia. The relative risk for South Africa was used along with country-specific HIV prevalence to split the general population mortality rate into HIV+ and HIV- rates, from which per-case probabilities were calculated.

## Influenza assumptions used in the study

It was assumed that in the absence of vaccination, 21.5% of individuals would be infected in each influenza season, either symptomatically or asymptomatically. This value is based on a three-year serological survey study in a setting without seasonal influenza vaccination (Vietnam, 2007-2010); no such data exists for South Africa ^17^. The symptomatic attack rate was assumed to be 5% based on a cohort study of Hayward et al. that used serology, weekly illness reporting, and RT-PCR identification of influenza from nasal swabs to estimate both infection and symptomatic illness, which found that approximately 75% of seasonal influenza infections were asymptomatic ^16^. The Hayward study found an average annual infection rate of 18%; however, this was for England where TIV coverage was approximately 20%, indicating that the no-vaccination infection rate is substantially higher. An alternative assumption of 10% symptomatic attack rate (with a 43% infection rate) was therefore considered in a sensitivity analysis.

Four co-circulating influenza strains were modelled: A(H3N2), A(H1N1), B Yamagata, and B Victoria. The transmissibility of each strain in each simulation year, which in the simulation model was represented by the probability of transmission due to pair-wise contact between infectious and susceptible individuals, was calibrated to match the proportion of each strain occurring that year for South Africa and Australia. South African strain data was supplied by the National Institute for Communicable Diseases, South Africa ^28^. Australian strain data was based on samples received at the WHO Collaborating Centre for Reference and Research on Influenza, Australia ^29, 30^. Calibration was conducted independently for each study year by iteratively adjusting the transmissibilities for the 4 strains until the attack rate was within 0.1% of the target (5%, or 10% for the higher attack rate sensitivity analysis scenario) and the proportion of each strain matched known ratios for the target year.

In South Africa, seasonal influenza vaccination coverage was less than 2%, thus strain transmissibilities for each year were calibrated so that a no-vaccination scenario gave a symptomatic attack rate of 5% and where the simulated strain ratios matched the observed South African strain ratios.

In Australia, vaccination coverage for the years studied averaged 20%, with 75% in those aged 65 and older, 11% in those under 65 ^31^. The strain transmissibilities for Australia were therefore calibrated so that (a) a no-vaccination simulation gave a symptomatic attack rate of 5%, to match the assumption made for South Africa, and (b) an additional simulation using *actual* Australian TIV vaccination practice (the 20% coverage mentioned above) matched the *observed* strain ratios. The resulting strain transmissibilities thus had the important properties that (a) the hypothetical scenario of no vaccination in Australia could be meaningfully compared with the no-vaccination scenario in South Africa, with both assuming a symptomatic attack rate of 5%, while at the same time (b) allowing per-case hospitalisation and mortality probabilities to be calculated using Australian data collected while vaccination was in use.

The proportion of influenza due to each of the two influenza B lineages observed in each country, along with the B lineage present in the TIV, is given for each study year in Table S3.

In order to gauge the relative importance of (a) strain circulation and (b) community structure in determining the effectiveness of QIV *versus* TIV, we repeated the main analysis using South African strain ratios in the Australian model, and Australian strain ratios in the South African model. In the latter case we took into account the fact that TIV was used in Australia during the study years i.e. we first calculated what the Australian no-vaccination strain ratios would have been without TIV, and transferred those to the South African model.

**Table S3 - Percentage of influenza B lineages and linage used in TIV**

| **Year** | **South Africa** | | | **Australia** | | |
| --- | --- | --- | --- | --- | --- | --- |
|  | TIV | Victoria | Yamagata | TIV | Victoria | Yamagata |
| 2003 | V | 0.0% | **7.0%** | V | 0.2% | **0.6%** |
| 2004 | V | 0.0% | **2.6%** | V | 4.2% | **20.8%** |
| 2005 | Y | **15.9%** | 2.9% | Y | **10.4%** | 11.1% |
| 2006 | V | 9.0% | **1.4%** | V | 32.9% | **1.5%** |
| 2007 | V | 14.5% | **11.7%** | V | 1.2% | **4.1%** |
| 2008 | Y | **3.3%** | 9.8% | Y | **32.4%** | 30.9% |
| 2009 | Y | **2.9%** | 3.1% | Y | **0.7%** | 0.5% |
| 2010 | V | 19.4% | **32.6%** | V | 11.5% | **1.2%** |
| 2011 | V | 3.8% | **5.9%** | V | 30.6% | **0.6%** |
| 2012 | V | 26.5% | **14.6%** | V | 30.1% | **3.3%** |
| 2013 | Y | **7.8%** | 8.8% | Y | **2.7%** | 34% |

For each community and study year, the percentage of circulating influenza due to the Victoria and Yamagata influenza B lineages (from South African ^28^ and Australian ^29, 30^ surveillance data is given, along with the lineage included the TIV for that community. Percentage for the lineage that was not included in the TIV is shown in bold face. Abbreviations: V – Victoria; Y – Yamagata.

It was assumed that symptomatic influenza infection lasted 6 days: 0.5 days latent; 1 day asymptomatic and partially infectious; 2 days symptomatic and fully infectious; followed by 2.5 days symptomatic and partially infectious ^18^. Partially infectious people were assumed to have 50% lower chance of transmitting influenza ^18^. It was assumed that while a person was infected with influenza (i.e. during the 6-day infection timeline described above), infection with another strain could not occur. Apart from this, no cross-strain protection from infection was assumed.

The long-term dynamics of a population’s influenza immune state is inherently complex. The proportion of the population immune to each strain each year depends on the immune state from the previous year, the amount of influenza transmission for each strain, antigenic drift of strains, possible cross-protection between strains, possible waning immunity, and vaccination ^32^. Rather than attempt to model the multi-year cause-and-effect relationship between these factors with a dynamic model that tracked each individual’s immune state over 11 years, we assumed that on average 27% of the population is immune to any given strain in any year ^19, 20^. That is, at the start of each year, each person is randomly determined to be immune with 27% probability, independently for each strain. Since we assume that 21.5% of the population is infected anew each year (symptomatically or asymptomatically), the assumption of 27% pre-existing immunity in each year is consistent with infection-acquired immunity lasting on average slightly longer than one year. In a sensitivity analysis we examine an alternative age distribution for this pre-existing immunity, assuming a greater number of older and less younger people being immune, but keeping the same overall 27%.

## Vaccine assumptions

Two different vaccines were modelled: trivalent (TIV), which contains two influenza A strains, and one B strain; and quadrivalent (QIV), which is effective against two A strains and two B lineages. In both models the TIV vaccine composition used in Australia was assumed and this was the WHO recommendation for the southern hemisphere for that year ^33^.

It was assumed that vaccine efficacy (VE) was 65% for individuals aged up to 65 years, and 55% in people aged 65+ years ^21-23, 34-37^. As different studies of vaccine efficacy report a range of VE values we conducted two sensitivity analyses. In one it was assumed that VE was significantly lower, particularly in older individuals, with VE 50% in ages less than 65 and 26% in ages 65+ ^36^. Several studies have indicated that vaccine efficacy may also vary according to influenza strain ^36-38^ ; we therefore also conducted a sensitivity analysis assuming that VE against pre-2009 A/H1N1 was 31%, VE against A/H1N1 post-2009 was 88%, VE against A/H3N2 was 52%, and VE against influenza B was 65% ^37^.

In the main scenarios analysed no cross-strain vaccine protection was assumed; i.e. vaccination with TIV did not provide any protection against the influenza B lineage not included in the vaccine. Results from recent studies indicate that vaccination against one influenza B strain may provide some protection against the other ^36, 38^. In order to examine this possibility a sensitivity analysis was performed with various levels of cross-strain protection.

It was also assumed that in 2009 neither TIV nor QIV provided protection against the dominant pandemic 2009 A/H1N1 strain, which replaced the previously circulating A/H1N1 strain.

We further assumed that immunity gained from vaccination would not decrease over a single influenza season. As there is evidence that vaccine-derived immunity does wane within a year ^39-41^, we conducted a sensitivity analysis assuming this feature.

The number of vaccine doses used in each year was equal to 2%, 5%, 15%, or 20% of the population (depending on the scenario), with vaccination prioritisation strategies described below. The vaccination programme was assumed to have been completed before the beginning of each simulated influenza season.

## Vaccination prioritisation

Four different vaccination prioritisation strategies were used for each vaccine (TIV and QIV), and together with a no-vaccination strategy give a total of 9 strategies. For targeted strategies 1, 2 and 3 below it was assumed that not all members of the target groups would be reached by the vaccination programme. 50% of the members of the highest priority group were chosen at random and as many as possible of that subgroup were vaccinated, as the vaccine supply allowed. If the vaccine supply permitted, 50% of the next highest priority group were targeted, and so on.

The prioritisation strategies were:

1. **Vulnerable age groups-first**. Vaccine was distributed first to those aged 65+, followed by those under 5, followed by adults in the 45-64 age group.
2. **HIV/Vulnerable-first.** Vaccine was first distributed to those who are HIV positive, and the remainder distributed according to the Vulnerable-first strategy.
3. **Transmitters-first.** The highest priority group were the 13-17 years of age adolescent cohort (Agincourt) or children aged 5-12 (Albany). Vaccine was distributed according to age cohorts, prioritising those who contributed most to transmission; that is, those with the highest age-specific attack rate in a situation where no vaccination occured.
4. **Randomised.** The vaccine was distributed randomly within the population.

The vulnerable-first strategy approximates the strategy employed in countries that extensively use TIV, where vaccine uptake is highest in the over 65 age group. The HIV/vulnerable-first strategy approximates the priority classes recommended by the WHO, though without prioritising vaccination of pregnant women ^42^. We note that there is some evidence that vaccine efficacy may be lower in young children and those with HIV ^43, 44^. This has not been included in the current model, and as a result the effectiveness of strategies 1 and 2 may be overestimated. The transmitters-first strategy is an alternative strategy that attempts to maximise the effect of vaccination by targeting those age groups most responsible for transmission and the resulting indirect herd immunity effects. While not a plausibly policy, the randomised strategy 4 is included as an unbiased baseline against which the other strategies may be compared.

## Health outcomes decision process

Output from the simulation models captures symptomatic influenza cases, categorised by age and HIV status. Based on this simulation output, an age-specific health outcomes process for each community was used to estimate the health burden of influenza on the community in terms of hospitalisation and death, as in Figure 1.

Age-specific hospitalisation probabilities for the Agincourt, South Africa model were based on the age-specific incidence of laboratory-confirmed influenza-associated lower respiratory tract infections in rural areas of South Africa during the period of 2009-2011 [4]. Hospitalization incidence rates per 100,000 persons for these three years were averaged and used to calculate hospitalisation probabilities per influenza case. Data from the age cohorts in data source ^8^ were interpolated to the 7 age cohorts used in the simulation model. To transform these rates to probabilities of hospitalisation visit per influenza case, the *age-specific population hospitalisation incidence rates* were divided by age-specific symptomatic influenza attack rates provided by the simulation model, as indicated in Figure 1. This captured the increased hospitalisation and mortality rates found in the very young and elderly following influenza infection, compared to the rest of the population.

We used the same methodology to generate age-specific hospitalisation probabilities for the Australian (Albany) model. Australian data on influenza-associated hospitalisation were used for the Australian model ^25^, and similar hospitalisation rates were assumed for influenza A and B ^45^.

Probabilities of influenza-associated mortality given symptomatic infection (i.e. the case fatality ratio) were derived for both communities in a similar way as for hospitalisations, using influenza mortality rate estimates rather than hospitalisation rate estimates ^3, 26, 27, 46, 47^.

The calculation of the case hospitalisation and case fatality probabilities for the Australian model differed in an important way from the South African calculations. In South Africa, no seasonal influenza vaccination was used in the period for which the hospitalisation and mortality data was collected, and therefore age-specific attack rates generated by a no-vaccination simulation were used to calculate hospitalisation and mortality probabilities. In Australia, TIV was extensively used during this period and therefore case hospitalisation and case fatality probabilities were calculated using age-specific attack rates generated using the Australian model with TIV vaccination occurring with coverage of 75% in individuals aged 65 years and older, and 11% in other age groups, as described in ^31^.

To account for the effect of health status on the probability of hospitalisation and death, age-specific relative risks of hospitalisation of HIV infected versus HIV uninfected individuals were used to obtain health status-specific hospitalisation and death probabilities [4]. Relative risks for hospitalisations and death from South Africa were applied to the Australian health outcome process, as no similar relative risk data were available for Australia. Further details, including the health outcome probabilities are contained in the Supporting Information.

## Simulation experiments

In addition to a no-vaccination scenario, 32 vaccination scenarios were simulated; for both TIV and QIV the four prioritisation strategies described above were simulated, each assuming 2%, 5%, 15% and 20% vaccination coverage. For each scenario, each influenza season from 2002-2013 (11 years) was simulated in both communities, giving a total of 704 influenza season simulations.

Starting at the beginning of an influenza season, *on each day of the simulation* one individual from the population was randomly selected and challenged with infection by one of the four strains (with an equal probability for each strain), modelling the introduction of multiple strains into the community. This mechanism simulates infection introduced into the community from travellers entering the community or infected residents returning to the community. All simulations were repeated 40 times with different random number sequences controlling the outcome of stochastic events, which include the location and strain of seeded individuals and the outcome of infective contact events, and the results averaged. Previous analysis of similar simulation models has shown that the 40-run mean of the attack rate is 95% certain to differ by less than 1.2% of the mean attack rate, relative to a much larger set of experiment repeats, as described in ^1^.

The simulations generated detailed data giving the breakdown of influenza infection by age group, locale of infection, influenza strain, and co-morbidity status of the individual. From these simulation results, the number of hospitalisations and deaths due to influenza were calculated using the health outcome probabilities for each community.

# Additional Results

**Table S4 – Estimated influenza attack rates by community and vaccination scenario**

| **Agincourt** | | | | | | | | | |
| --- | --- | --- | --- | --- | --- | --- | --- | --- | --- |
|  | **Attack Rate (%)** | | | | | | | | |
| **Priority Strategies** | **no_Vacc** | **VC-2%** | | **VC-5%** | | **VC-15%** | | **VC-20%** | |
|  |  | TIV | QIV | TIV | QIV | TIV | QIV | TIV | QIV |
| **Random** | 4.9  (4.7 – 5.1) | 4.3  (4.1 – 4.6) | 4.3  (4.0 – 4.5) | 3.6  (3.4 – 3.8) | 3.4  (3.1 – 3.6) | 1.7  (1.5 – 1.9) | 1.3  (1.1 – 1.5) | 1.3  (1.1 – 1.5) | 0.8  (0.7 – 1.0) |
| **Trans** | 4.9  (4.7 – 5.1) | 4.0  (3.8 – 4.2) | 3.8  (3.6 – 4.1) | 2.8  (2.6 – 3.1) | 2.6  (2.4 – 2.9) | 1.4  (1.3 – 1.6) | 0.9  (0.8 – 1.1) | 1.2  (1.0 – 1.4) | 0.7  (0.6 – 0.8) |
| **Vuln** | 4.9  (4.7 – 5.1) | 4.7  (4.4 – 4.9) | 4.6  (4.4 – 4.9) | 4.3  (4.0 – 4.5) | 4.1  (3.9 – 4.4) | 2.8  (2.5 – 3.0) | 2.5  (2.3 – 2.8) | 2.0  (1.8 – 2.2) | 1.6  (1.4 – 1.8) |
| **H-Vuln** | 4.9  (4.7 – 5.1) | 4.2  (4.0 – 4.5) | 4.2  (4.0 – 4.5) | 3.6  (3.4 – 3.8) | 3.4  (3.2 – 3.6) | 2.5  (2.3 – 2.7) | 2.2  (1.9 – 2.4) | 1.9  (1.7 – 2.1) | 1.5  (1.3 – 1.7) |
| **Albany** | | | | | | | | | |
|  | **Attack Rate (%)** | | | | | | | | |
| **Priority Strategies** | **no_Vacc** | **VC-2%** | | **VC-5%** | | **VC-15%** | | **VC-20%** | |
|  |  | TIV | QIV | TIV | QIV | TIV | QIV | TIV | QIV |
| **Random** | 4.9  (4.6 – 5.2) | 4.2  (3.9 – 4.5) | 4.2  (3.9 – 4.5) | 3.3  (3.0 – 3.6) | 3.3  (3.0 – 3.6) | 1.3  (1.1 – 1.5) | 1.2  (1.0 – 1.4) | 0.9  (0.7 – 1.0) | 0.9  (0.7 – 1.0) |
| **Trans** | 4.9  (4.6 – 5.2) | 3.9  (3.6 – 4.2) | 4.0  (3.7 – 4.3) | 2.2  (1.9 – 2.4) | 2.1  (1.9 – 2.4) | 0.7  (0.6 – 0.8) | 0.6  (0.5 – 0.7) | 0.6  (0.5 – 0.7) | 0.5  (0.4 – 0.6) |
| **Vuln** | 4.9  (4.6 -5.2) | 4.7  (4.4 – 5.0) | 4.7  (4.5 – 5.0) | 4.6  (4.2 – 4.9) | 4.6  (4.3 – 4.9) | 2.6  (2.3 – 2.9) | 2.6  (2.3 – 2.9) | 2.1  (1.8 – 2.3) | 2.0  (1.7 – 2.2) |
| **H-Vuln** | 4.9  (4.6 -5.2) | 4.7  (4.4 – 5.0) | 4.7  (4.4 – 5.0) | 4.5  (4.2 – 4.8) | 4.5  (4.2 – 4.8) | 2.6  (2.3 – 2.9) | 2.5  (2.3 – 2.8) | 2.0  (1.8 – 2.3) | 1.9  (1.6 – 2.1) |

The results give the symptomatic attack rates as a percentage of the population averaged over 11 years, with 95% confidence interval given in parentheses. Abbreviations: VC – vaccination coverage (% of population); NV – no vaccination; Random – vaccine randomly distributed in population; Trans – transmitters-first vaccine prioritisation; Vuln – vulnerable-first vaccine prioritisation; H-Vuln comorbidity-then-vulnerable-first vaccine prioritisation.

**Table S5** - **Estimated respiratory infection hospitalisations due to influenza by community and TIV vaccination scenario**

| **Agincourt** | | | | | | | | | |
| --- | --- | --- | --- | --- | --- | --- | --- | --- | --- |
|  | **Hospitalisation per 100,000** | | | | | | | | |
| **Priority Strategies** | **no_Vacc** | **VC-2%** | | **VC-5%** | | **VC-15%** | | **VC-20%** | |
|  |  | TIV | QIV | TIV | QIV | TIV | QIV | TIV | QIV |
| **Random** | **60.4** | 53.5 | 52.4 | 44.1 | 41.1 | 20.9 | 16.0 | 16.0 | 10.3 |
| **Trans** | **60.4** | 50.1 | 48.4 | 36.1 | 34.4 | 18.8 | 12.9 | 15.2 | 9.1 |
| **Vuln** | **60.4** | 57.4 | 56.9 | 50.5 | 48.4 | 29.7 | 26.2 | 22.2 | 16.5 |
| **H-Vuln** | **60.4** | 51.1 | 50.5 | 41.7 | 38.6 | 25.8 | 21.0 | 19.9 | 14.7 |
| **Albany** | | | | | | | | | |
|  | **Hospitalisation per 100,000** | | | | | | | | |
| **Priority Strategies** | **no_Vacc** | **VC-2%** | | **VC-5%** | | **VC-15%** | | **VC-20%** | |
|  |  | TIV | QIV | TIV | QIV | TIV | QIV | TIV | QIV |
| **Random** | 215.5 | 182.7 | 182.6 | 144.5 | 144.4 | 57.0 | 54.0 | 38.4 | 37.9 |
| **Trans** | 215.5 | 174.0 | 175.3 | 99.4 | 97.7 | 33.1 | 29.7 | 28.6 | 24.5 |
| **Vuln** | 215.5 | 201.3 | 201.8 | 177.7 | 179.3 | 91.2 | 91.1 | 73.1 | 69.8 |
| **H-Vuln** | 215.5 | 200.0 | 200.1 | 175.7 | 175.8 | 91.5 | 89.4 | 72.6 | 66.3 |
| **Actual** |  |  |  |  |  |  |  | **55.8** |  |

Result values are estimated pneumonia influenza hospitalisations per 100,000 averaged over 11 years. The bold type values are results derived from calibrating the simulation model to hospitalisation data. Abbreviations: VC – vaccination coverage (% of population); NV – no vaccination; Random – vaccine randomly distributed in population; Trans – transmitters-first vaccine prioritisation; Vuln – vulnerable-first vaccine prioritisation; H-Vuln comorbidity-then-vulnerable-first vaccine prioritisation; Actual – Australian vaccination coverage (75% in ages 65+, 10% in ages 64 and less).

**Table S6 - Estimated mortality due to influenza by community and vaccination scenario**

| **Agincourt** | | | | | | | | | |
| --- | --- | --- | --- | --- | --- | --- | --- | --- | --- |
|  | **Deaths per 100,000** | | | | | | | | |
| **Priority Strategies** | **no_Vacc** | **VC-2%** | | **VC-5%** | | **VC-15%** | | **VC-20%** | |
|  |  | TIV | QIV | TIV | QIV | TIV | QIV | TIV | QIV |
| **Random** | **26.1** | 23.1 | 22.7 | 19.1 | 17.8 | 9.0 | 7.0 | 6.9 | 4.4 |
| **Trans** | **26.1** | 21.2 | 20.4 | 14.9 | 13.9 | 7.7 | 5.2 | 6.7 | 4.1 |
| **Vuln** | **26.1** | 22.1 | 21.6 | 19.3 | 18.2 | 12.4 | 10.9 | 9.5 | 7.0 |
| **H-Vuln** | **26.1** | 22.2 | 22.1 | 18.5 | 17.2 | 11.2 | 9.2 | 8.5 | 6.4 |
| **Albany** | | | | | | | | | |
|  | **Deaths per 100,000** | | | | | | | | |
| **Priority Strategies** | **no_Vacc** | **VC-2%** | | **VC-5%** | | **VC-15%** | | **VC-20%** | |
|  |  | TIV | QIV | TIV | QIV | TIV | QIV | TIV | QIV |
| **Random** | 11.6 | 9.8 | 9.9 | 7.7 | 7.7 | 3.1 | 2.9 | 2.1 | 2.0 |
| **Trans** | 11.6 | 9.4 | 9.4 | 5.4 | 5.3 | 1.8 | 1.7 | 1.6 | 1.3 |
| **Vuln** | 11.6 | 10.7 | 10.8 | 9.2 | 9.2 | 4.7 | 4.7 | 3.8 | 3.6 |
| **H-Vuln** | 11.6 | 10.7 | 10.7 | 9.1 | 9.0 | 4.7 | 4.6 | 3.8 | 3.5 |
| **Actual** |  |  |  |  |  |  |  | **3.0** |  |

Result values are estimated deaths attributable to influenza per 100,000 averaged over 11 years. The bold type values are results derived from calibrating the simulation model to influenza excess mortality estimates. Abbreviations: VC – vaccination coverage (% of population); NV – no vaccination; Random – vaccine randomly distributed in population; Trans – transmitters-first vaccine prioritisation; Vuln – vulnerable-first vaccine prioritisation; H-Vuln comorbidity-then-vulnerable-first vaccine prioritisation; Actual – Australian vaccination coverage (75% in ages 65+, 10% in ages 64 and less).

For Tables S5 and S6, result values are estimated hospitalisations and deaths attributable to influenza per 100,000 population averaged over 11 years. Vaccination coverage is 15%. Abbreviations: NV – no vaccination; TIV – trivalent influenza vaccine; QIV – quadrivalent influenza vaccine; (NV – TIV) hospitalisations or deaths averted due to TIV compared to no vaccination; (TIV – QIV) hospitalisations or deaths averted due to QIV compared to TIV; Random – vaccine randomly distributed in population; Trans – transmitters-first vaccine prioritisation; Vuln – vulnerable-first vaccine prioritisation; H-Vuln comorbidity-then-vulnerable-first vaccine prioritisation.

**Table S7 – Attack rates for Agincourt, transmitters-first, coverage 15%**

| **Year** | **NV** | **TIV** | **QIV** | **NV-TIV** | **TIV-QIV** | **B lineage used in TIV** | **mismatch %** |
| --- | --- | --- | --- | --- | --- | --- | --- |
| 2003 | 4.9 | 1.4 | 1.0 | 3.5 | 0.4 | Victoria | 7.0 |
| 2004 | 4.7 | 1.0 | 0.9 | 3.7 | 0.1 | Victoria | 2.6 |
| 2005 | 4.6 | 1.5 | 0.6 | 3.1 | 0.9 | Yamagata | 15.9 |
| 2006 | 5.1 | 1.0 | 0.9 | 4.1 | 0.1 | Victoria | 1.4 |
| 2007 | 5.0 | 1.1 | 0.6 | 3.8 | 0.6 | Victoria | 11.7 |
| 2008 | 5.1 | 0.9 | 0.8 | 4.2 | 0.1 | Yamagata | 3.3 |
| 2009 | 5.0 | 2.8 | 2.8 | 2.1 | 0.1 | Yamagata | 2.9 |
| 2010 | 4.9 | 2.6 | 0.5 | 2.3 | 2.0 | Victoria | 32.6 |
| 2011 | 4.9 | 0.9 | 0.7 | 4.0 | 0.2 | Victoria | 5.9 |
| 2012 | 4.7 | 1.6 | 0.7 | 3.1 | 0.9 | Victoria | 14.6 |
| 2013 | 5.0 | 1.0 | 0.8 | 4.0 | 0.3 | Yamagata | 7.8 |
| Average | 4.9 | 1.4 | 0.9 | 3.5 | 0.5 |  | 9.61 |

**Table S8 – Attack rates for Albany, transmitters-first, coverage 15%**

| **Year** | **NV** | **TIV** | **QIV** | **NV-TIV** | **TIV-QIV** | **B lineage used in TIV** | **mismatch %** |
| --- | --- | --- | --- | --- | --- | --- | --- |
| 2003 | 4.8 | 0.4 | 0.4 | 4.5 | 0.0 | Victoria | 0.6 |
| 2004 | 4.9 | 0.9 | 0.5 | 4.0 | 0.5 | Victoria | 20.8 |
| 2005 | 5.1 | 0.5 | 0.4 | 4.5 | 0.2 | Yamagata | 10.4 |
| 2006 | 4.9 | 0.3 | 0.3 | 4.5 | 0.0 | Victoria | 1.5 |
| 2007 | 4.9 | 0.4 | 0.3 | 4.6 | 0.0 | Victoria | 4.1 |
| 2008 | 4.5 | 0.8 | 0.4 | 3.7 | 0.5 | Yamagata | 32.4 |
| 2009 | 5.1 | 2.8 | 2.9 | 2.3 | -0.1 | Yamagata | 0.7 |
| 2010 | 4.7 | 0.4 | 0.4 | 4.3 | 0.0 | Victoria | 1.2 |
| 2011 | 5.2 | 0.4 | 0.3 | 4.8 | 0.1 | Victoria | 0.6 |
| 2012 | 4.8 | 0.4 | 0.4 | 4.5 | 0.0 | Victoria | 3.3 |
| 2013 | 5.1 | 0.4 | 0.4 | 4.7 | 0.0 | Yamagata | 2.7 |
| Average | 4.9 | 0.7 | 0.6 | 4.2 | 0.1 |  | **7.21** |

**Table S9 – Advantage of QIV vs TIV for combinations of community model and influenza circulation**

|  | | *community model* | |
| --- | --- | --- | --- |
|  |  | **AUS** | **SA** |
| *strain ratios* | **AUS** | 0.1% | 0.1% |
|  | **SA** | 0.5% | 0.4% |

Table shows the percentage of the population that would avoid infection using QIV compared to TIV for the transmitters-first strategy, with 15% vaccination coverage, for all combinations of community model and influenza strain ratios.

**Table S10 - Age breakdown of hospitalisation and death per 100,000 population in Agincourt** **for 15% vaccination coverage**

|  | **Agincourt** | | | | | | |
| --- | --- | --- | --- | --- | --- | --- | --- |
| **Strategy** | **Age Groups** | **Hospitalisations per 100,000** | | | **Deaths per 100,000** | | |
|  |  | **NV** | **TIV** | **QIV** | **NV** | **TIV** | **QIV** |
| **Trans** | **(0-5)** | 30.1 | 9.8 | 6.9 | 1.4 | 0.5 | 0.3 |
|  | **(6-12)** | 3.2 | 0.8 | 0.5 | 0.4 | 0.1 | 0.1 |
|  | **(13- 17)** | 2.4 | 0.6 | 0.3 | 0.3 | 0.1 | 0.0 |
|  | **(18-24)** | 2.8 | 0.9 | 0.6 | 0.6 | 0.2 | 0.1 |
|  | **(25-44)** | 13.1 | 4.1 | 2.8 | 3.2 | 1.0 | 0.7 |
|  | **(45-64)** | 6.5 | 1.9 | 1.3 | 4.9 | 1.5 | 1.0 |
|  | **(65+)** | 2.3 | 0.7 | 0.5 | 15.4 | 4.5 | 3.0 |
|  | **Overall** | **60.4** | **18.8** | **12.9** | **26.1** | **7.7** | **5.2** |
| **H-Vuln** | **(0-5)** | 30.1 | 11.8 | 9.2 | 1.4 | 0.6 | 0.4 |
|  | **(6-12)** | 3.2 | 1.6 | 1.4 | 0.4 | 0.2 | 0.1 |
|  | **(13- 17)** | 2.4 | 1.2 | 1.1 | 0.3 | 0.1 | 0.1 |
|  | **(18-24)** | 2.8 | 1.4 | 1.2 | 0.6 | 0.3 | 0.2 |
|  | **(25-44)** | 13.1 | 5.7 | 4.7 | 3.2 | 1.4 | 1.1 |
|  | **(45-64)** | 6.5 | 3.1 | 2.7 | 4.9 | 2.4 | 2.1 |
|  | **(65+)** | 2.3 | 1.0 | 0.8 | 15.4 | 6.3 | 5.1 |
|  | **Overall** | **60.4** | **25.8** | **21.0** | **26.1** | **11.2** | **9.2** |

**Table S11 - Age breakdown of hospitalisation and death per 100,000 population in Albany** **for 15% vaccination coverage**

|  | **Albany** | | | | | | |
| --- | --- | --- | --- | --- | --- | --- | --- |
| **Strategy** | **Age Groups** | **Hospitalisations per 100,000** | | | **Deaths per 100,000** | | |
|  |  | **NV** | **TIV** | **QIV** | **NV** | **TIV** | **QIV** |
| **Trans** | **(0-5)** | 21.4 | 2.7 | 2.2 | 0.2 | 0.0 | 0.0 |
|  | **(6-12)** | 3.5 | 0.4 | 0.4 | 0.0 | 0.0 | 0.0 |
|  | **(13- 17)** | 3.6 | 0.5 | 0.4 | 0.0 | 0.0 | 0.0 |
|  | **(18-24)** | 3.8 | 0.6 | 0.5 | 0.0 | 0.0 | 0.0 |
|  | **(25-44)** | 13.8 | 2.1 | 1.9 | 0.2 | 0.0 | 0.0 |
|  | **(45-64)** | 20.9 | 3.2 | 2.9 | 0.4 | 0.1 | 0.0 |
|  | **(65+)** | 148.5 | 23.5 | 21.4 | 10.7 | 1.7 | 1.5 |
|  | **Overall** | **215.5** | **33.1** | **29.7** | **11.6** | **1.8** | **1.6** |
| **H-Vuln** | **(0-5)** | 21.4 | 8.0 | 7.6 | 0.2 | 0.1 | 0.1 |
|  | **(6-12)** | 3.5 | 2.1 | 2.0 | 0.0 | 0.0 | 0.0 |
|  | **(13- 17)** | 3.6 | 2.1 | 2.1 | 0.0 | 0.0 | 0.0 |
|  | **(18-24)** | 3.8 | 2.0 | 2.0 | 0.0 | 0.0 | 0.0 |
|  | **(25-44)** | 13.8 | 7.4 | 7.3 | 0.2 | 0.1 | 0.1 |
|  | **(45-64)** | 20.9 | 10.3 | 10.2 | 0.4 | 0.2 | 0.2 |
|  | **(65+)** | 148.5 | 59.7 | 58.2 | 10.7 | 4.3 | 4.2 |
|  | **Overall** | **215.5** | **91.5** | **89.4** | **11.6** | **4.7** | **4.6** |

# Sensitivity Analyses

Sensitivity analyses were conducted over key influenza and vaccine model parameters to determine how alternative assumptions affected seasonal influenza vaccination outcomes. The impact that these alternative assumptions have on the effectiveness of vaccination in reducing influenza attack rates, and particularly any impact on the relative effectiveness of TIV vs QIV, are summarised below. Unless otherwise stated, vaccination coverage for the sensitivity analysis scenarios was 15%.

## Vaccination B-lineage cross protection: Tables S12 and S13

Tables S12 and S13 provide further hospitalisation and mortality results for the vaccination B-lineage cross-protection sensitivity analysis, which is described in the main manuscript section 3.4.

## Vaccine efficacy: Tables S14 to S17

Vaccine efficacy of 65% for age groups less than 65 and 55% for the 65 plus age group was assumed in the main analysis scenarios. We performed sensitivity analysis for two alternative vaccine efficacy assumptions. Firstly, we assumed a lower vaccine efficacy of 50% for the under-65 age groups and 26% for the 65+ age group, based on data taken from ^36^. As might be expected, this rendered all vaccination strategies less effective. For example, 15% TIV with the HIV/vulnerable-first strategy in the South African community reduced the attack rate from 4.9% to 2.8%, compared to 2.5% for the main analysis VE assumptions.

Secondly, we assumed subtype-specific vaccine efficacy as follows: VE for pre-2009 A/H1N1 of 31%, VE for post-2009 A/H1N1 of 88%, VE for A/H3N2 of 52% and VE for influenza B of 65%); these data being based on an Australian study ranging over 5 years (2007 - 2011) ^37^. Although the post-2009 A/H1N1 VE is higher than the main analysis assumption of 65%, VE for other strains are lower than 65%, and the result is similar to the results for the lower-VE assumption described above, although less pronounced.

## Higher 40% vaccination coverage: Tables S18 and S19

In order to determine the effect of very high influenza coverage rates, we examined a scenario where vaccination coverage was 40% - which is higher than either Australia or South Africa.

## Alternative symptomatic attack rate: Tables S20 and S21

In deriving the main results a no-vaccination symptomatic attack rate of 5% was assumed with a 21.5% infection rate. Since the size of annual seasonal influenza epidemics vary year to year and country to country, we further examined a higher no-vaccination symptomatic attack rate of 10%, and infection rate of 43%, over the 11 influenza seasons considered in this study.

The effect of this alternative assumption was that vaccination resulted in a larger *absolute* reduction in the attack rate, but that the *relative* reduction in the attack rate was smaller. For example with the main analysis assumptions in the South African community, 15% TIV with the HIV/vulnerable-first strategy reduced the attack rate from 4.9% to 2.5%, an absolute reduction of 2.4% of the population and a relative reduction of 49% of the no-vaccination attack rate. With the higher no-vaccination infection and attack rates, the same strategy reduced the attack rate from 10.1% to 7.2%, an absolute reduction of 2.9% but a relative reduction of 29%. The smaller relative reduction is due to the higher level of influenza transmission, which reduces the indirect herd immunity effect due to vaccination.

## Waning immunity: Tables S22 and S23

In the main analysis, we assumed immunity gained from seasonal influenza vaccination would not wane during the influenza season. We examined an alternative assumption that immunity gained from vaccination would decrease over the year following vaccination. Successfully vaccinated individuals, i.e. those who became immune due to vaccination, were marked with the vaccine strains to which he/she was immune, with an immunity level starting at 100% (i.e. fully protected against infection). It was assumed that 3 months after successful vaccination a vaccine-immune individual would become partially (25%) susceptible due to waning immunity (i.e. the probability of infection was 25% of the un-vaccinated probability). After 6 months there would be a 50% reduction in immunity, and after 9 months a 75% reduction in immunity. Finally, after 1 year vaccine-induced immunity had waned to zero. These assumptions regarding waning immunity were based on recent findings of ^39-41^.

The results of this sensitivity analysis indicated that attack rate reductions were significantly smaller if immunity gained from vaccines waned during the influenza season. Using the South African community with 15% TIV and the HIV/vulnerable-first strategy again as an example, if the waning immunity assumption is made, vaccination reduces the attack rate from 4.9% to 3.7%, rather than from 4.9% to 2.5% under the no-waning-immunity assumption.

## Pre-existing immunity: Tables S24 and S25

In the main results, we assumed 27% of all individuals across all age groups had pre-existing immunity in each year. Several longitudinal influenza studies have found that individuals can experience infection by the same influenza subtype in consecutive influenza seasons, and that this is more common in children than older individuals ^48-50^. In order to examine the possibility that children have a higher rate of immunity loss, we performed a sensitivity analysis where we assumed that individuals aged <18 years have *half* the probability of having pre-existing immunity compared to 18+ aged individuals, but retaining the overall pre-existing immunity of 27% in the total population. Using the age demographics of the South African community we calculated that this assumption results in ~17.7% of individuals aged < 18 years having pre-existing immunity and ~35.5% of individuals aged 18+ years having pre-existing immunity at the beginning of each simulated influenza season. Similarly in Australia, ~15.7% of individuals aged < 18 years had pre-existing immunity and ~31.4% of individuals aged 18+ years had pre-existing immunity at the beginning of each season.

The effect of this alternative assumption regarding age distribution of pre-existing immunity was to make the vulnerable-first prioritisation strategies slightly less effective, but the transmitters-first strategy slightly more effective. For example, using the South African community with 15% TIV as an example, under the altered pre-existing immunity assumption the HIV/vulnerable-first strategy reduced the attack rate from 5% to 2.7% rather than 2.5% with the original assumption, whereas the transmitters-first strategy with the altered assumption reduces the attack rate from 5% to 1.3% compared to 1.4% with the original assumption. This is due to transmission occurring at (slightly) higher levels in the younger age groups (due to their lower pre-existing immunity) and that these are the age groups targeted by the transmitters-first strategy.

**Hospitalisations and mortality numbers for different sensitivity analyses scenarios**

**Table S12 - Hospitalisation per 100,000 population in Agincourt and Albany** **for 15% vaccination coverage and for different levels of cross protection**

| **Priority Strategy** | **No vaccination** | **TIV** | | | | | **QIV** |
| --- | --- | --- | --- | --- | --- | --- | --- |
|  |  | **0% VE against non TIV B-lineage** | **7% VE against non TIV B-lineage** | **13% VE against non TIV B-lineage** | **26% VE against non TIV B-lineage** | **52% VE against non TIV B-lineage** |  |
| ***Agincourt*** | | | | | | | |
| **Random** | 60.4 | 20.9 | 18.0 | 16.6 | 16.0 | 15.6 | 16.0 |
| **Trans** | 60.4 | 18.8 | 15.9 | 13.6 | 13.3 | 12.4 | 12.9 |
| **Vuln** | 60.4 | 29.7 | 27.4 | 25.8 | 25.3 | 25.3 | 26.1 |
| **H-Vuln** | 60.4 | 25.8 | 23.6 | 21.0 | 20.5 | 20.8 | 21.0 |
| ***Albany*** | | | | | | | |
| **Random** | 215.5 | 57.0 | 56.6 | 56.4 | 55.9 | 55.2 | 54.0 |
| **Trans** | 215.5 | 32.3 | 28.9 | 29.9 | 29.0 | 29.0 | 29.7 |
| **Vuln** | 215.5 | 91.2 | 91.5 | 91.0 | 91.2 | 91.9 | 91.1 |
| **H-Vuln** | 215.5 | 91.5 | 91.8 | 91.2 | 91.9 | 90.4 | 89.4 |

**Table S13 - Deaths per 100,000 population in Agincourt and Albany** **for 15% vaccination coverage and for different levels of cross protection**

| **Priority Strategy** | **No vaccination** | **TIV** | | | | | **QIV** |
| --- | --- | --- | --- | --- | --- | --- | --- |
|  |  | **0% VE against non TIV B-lineage** | **7% VE against non TIV B-lineage** | **13% VE against non TIV B-lineage** | **26% VE against non TIV B-lineage** | **52% VE against non TIV B-lineage** |  |
| ***Agincourt*** | | | | | | | |
| **Random** | 26.1 | 9.0 | 7.9 | 7.1 | 6.9 | 6.7 | 7.0 |
| **Trans** | 26.1 | 7.7 | 6.2 | 5.5 | 5.4 | 5.0 | 5.2 |
| **Vuln** | 26.1 | 12.4 | 11.6 | 10.8 | 10.6 | 10.6 | 10.9 |
| **H-Vuln** | 26.1 | 11.2 | 10.0 | 9.2 | 9.0 | 9.1 | 9.2 |
| ***Albany*** | | | | | | | |
| **Random** | 11.6 | 3.1 | 3.2 | 3.1 | 3.2 | 3.0 | 2.9 |
| **Trans** | 11.6 | 1.8 | 1.7 | 1.7 | 1.6 | 1.6 | 1.7 |
| **Vuln** | 11.6 | 4.7 | 4.7 | 4.7 | 4.7 | 4.8 | 4.7 |
| **H-Vuln** | 11.6 | 4.7 | 4.7 | 4.8 | 4.7 | 4.7 | 4.6 |

**Table S14 - Hospitalisation per 100,000 population in Agincourt and Albany** **for 15% vaccination coverage and for lower vaccine effectiveness (i.e. 51% for <65 age groups and 26% for 65+ age groups)**

| **Priority Strategy** | **No vaccination** | **TIV** | **QIV** |
| --- | --- | --- | --- |
| ***Agincourt*** | | | |
| **Random** | 60.4 | 27.1 | 22.5 |
| **Trans** | 60.4 | 23.8 | 18.7 |
| **Vuln** | 60.4 | 35.1 | 31.7 |
| **H-Vuln** | 60.4 | 31.6 | 27.7 |
| ***Albany*** | | | |
| **Random** | 215.5 | 85.6 | 81.4 |
| **Trans** | 215.5 | 42.8 | 39.8 |
| **Vuln** | 215.5 | 125.0 | 124.4 |
| **H-Vuln** | 215.5 | 122.2 | 124.9 |

**Table S15 - Deaths per 100,000 population in Agincourt and Albany** **for 15% vaccination coverage and for lower vaccine effectiveness (i.e. 51% for <65 age groups and 26% for 65+ age groups)**

| **Priority Strategy** | **No vaccination** | **TIV** | **QIV** |
| --- | --- | --- | --- |
| ***Agincourt*** | | | |
| **Random** | 26.1 | 11.9 | 9.9 |
| **Trans** | 26.1 | 9.9 | 7.6 |
| **Vuln** | 26.1 | 15.7 | 14.2 |
| **H-Vuln** | 26.1 | 14.5 | 13.0 |
| ***Albany*** | | | |
| **Random** | 11.6 | 4.6 | 4.4 |
| **Trans** | 11.6 | 2.4 | 2.2 |
| **Vuln** | 11.6 | 6.7 | 6.6 |
| **H-Vuln** | 11.6 | 6.6 | 6.7 |

**Table S16 - Hospitalisation per 100,000 population in Agincourt and Albany** **for 15% vaccination coverage and for subtype specific vaccine effectiveness**

| **Priority Strategy** | **No vaccination** | **TIV** | **QIV** |
| --- | --- | --- | --- |
| ***Agincourt*** | | | |
| **Random** | 60.4 | 28.8 | 23.3 |
| **Trans** | 60.4 | 25.6 | 20.1 |
| **Vuln** | 60.4 | 36.2 | 32.6 |
| **H-Vuln** | 60.4 | 33.1 | 28.6 |
| ***Albany*** | | | |
| **Random** | 215.5 | 88.5 | 73.4 |
| **Trans** | 215.5 | 60.1 | 41.0 |
| **Vuln** | 215.5 | 124.7 | 115.0 |
| **H-Vuln** | 215.5 | 124.4 | 112.3 |

**Table S17 - Deaths per 100,000 population in Agincourt and Albany** **for 15% vaccination coverage and for subtype specific vaccine effectiveness**

| **Priority Strategy** | **No vaccination** | **TIV** | **QIV** |
| --- | --- | --- | --- |
| ***Agincourt*** | | | |
| **Random** | 26.1 | 12.4 | 10.0 |
| **Trans** | 26.1 | 10.7 | 8.3 |
| **Vuln** | 26.1 | 15.5 | 13.8 |
| **H-Vuln** | 26.1 | 14.5 | 12.5 |
| ***Albany*** | | | |
| **Random** | 11.6 | 4.8 | 4.0 |
| **Trans** | 11.6 | 3.3 | 2.3 |
| **Vuln** | 11.6 | 6.6 | 6.0 |
| **H-Vuln** | 11.6 | 6.6 | 5.9 |

**Table S18 - Hospitalisation per 100,000 population in Agincourt and Albany** **for 40% vaccination coverage**

| **Priority Strategy** | **No vaccination** | **TIV** | **QIV** |
| --- | --- | --- | --- |
| ***Agincourt*** | | | |
| **Random** | 60.4 | 12.6 | 5.7 |
| **Trans** | 60.4 | 11.9 | 5.7 |
| **Vuln** | 60.4 | 12.6 | 6.4 |
| **H-Vuln** | 60.4 | 12.2 | 5.9 |
| ***Albany*** | | | |
| **Random** | 215.5 | 26.9 | 23.5 |
| **Trans** | 215.5 | 24.2 | 21.8 |
| **Vuln** | 215.5 | 28.7 | 24.7 |
| **H-Vuln** | 215.5 | 28.8 | 25.1 |

**Table S19 - Deaths per 100,000 population in Agincourt and Albany** **for 40% vaccination coverage**

| **Priority Strategy** | **No vaccination** | **TIV** | **QIV** |
| --- | --- | --- | --- |
| ***Agincourt*** | | | |
| **Random** | 26.1 | 5.4 | 2.5 |
| **Trans** | 26.1 | 5.3 | 2.8 |
| **Vuln** | 26.1 | 5.3 | 2.7 |
| **H-Vuln** | 26.1 | 5.2 | 2.5 |
| ***Albany*** | | | |
| **Random** | 11.6 | 1.5 | 1.3 |
| **Trans** | 11.6 | 1.3 | 1.2 |
| **Vuln** | 11.6 | 1.5 | 1.3 |
| **H-Vuln** | 11.6 | 1.5 | 1.3 |

**Table S20 - Hospitalisation per 100,000 population in Agincourt and Albany** **for 15% vaccination coverage and 10% symptomatic attack rate**

| **Priority Strategy** | **No vaccination** | **TIV** | **QIV** |
| --- | --- | --- | --- |
| ***Agincourt*** | | | |
| **Random** | 60.3 | 36.0 | 30.4 |
| **Trans** | 60.3 | 36.1 | 29.7 |
| **Vuln** | 60.3 | 39.6 | 35.1 |
| **H-Vuln** | 60.3 | 35.7 | 30.6 |
| ***Albany*** | | | |
| **Random** | 127.6 | 67.7 | 64.7 |
| **Trans** | 127.6 | 45.1 | 40.3 |
| **Vuln** | 127.6 | 76.4 | 74.9 |
| **H-Vuln** | 127.6 | 76.8 | 74.4 |

**Table S21 - Deaths per 100,000 population in Agincourt and Albany** **for 15% vaccination coverage and 10% symptomatic attack rate**

| **Priority Strategy** | **No vaccination** | **TIV** | **QIV** |
| --- | --- | --- | --- |
| ***Agincourt*** | | | |
| **Random** | 26.1 | 15.5 | 13.1 |
| **Trans** | 26.1 | 14.9 | 12.1 |
| **Vuln** | 26.1 | 16.5 | 14.5 |
| **H-Vuln** | 26.1 | 15.5 | 13.2 |
| ***Albany*** | | | |
| **Random** | 7.1 | 3.7 | 3.5 |
| **Trans** | 7.1 | 2.5 | 2.3 |
| **Vuln** | 7.1 | 4.0 | 3.9 |
| **H-Vuln** | 7.1 | 4.1 | 3.9 |

**Table S22 - Hospitalisation per 100,000 population in Agincourt and Albany** **for 15% vaccination coverage and waning immunity scenario**

| **Priority Strategy** | **No vaccination** | **TIV** | | **QIV** | |
| --- | --- | --- | --- | --- | --- |
|  |  | **no Waning Immunity** | **Immunity wanes within a year** | **no**  **Waning Immunity** | **Immunity wanes within a year** |
| ***Agincourt*** | | | | | |
| **Random** | 60.4 | 20.9 | 38.9 | 16.0 | 35.8 |
| **Trans** | 60.4 | 18.8 | 35.2 | 12.9 | 30.8 |
| **Vuln** | 60.4 | 29.7 | 45.0 | 26.1 | 42.9 |
| **H-Vuln** | 60.4 | 25.8 | 42.1 | 21.0 | 39.4 |
| ***Albany*** | | | | | |
| **Random** | 215.5 | 60.3 | 127.0 | 57.9 | 124.9 |
| **Trans** | 215.5 | 33.5 | 82.5 | 30.1 | 82.8 |
| **Vuln** | 215.5 | 94.4 | 155.5 | 94.4 | 159.6 |
| **H-Vuln** | 215.5 | 94.2 | 157.4 | 93.1 | 161.8 |

**Table S23 - Deaths per 100,000 population in Agincourt and Albany** **for 15% vaccination coverage and waning immunity scenario**

| **Priority Strategy** | **No vaccination** | **TIV** | | **QIV** | |
| --- | --- | --- | --- | --- | --- |
|  |  | **no Waning Immunity** | **Immunity wanes within a year** | **no**  **Waning Immunity** | **Immunity wanes within a year** |
| ***Agincourt*** | | | | | |
| **Random** | 26.1 | 9.0 | 16.7 | 7.0 | 15.4 |
| **Trans** | 26.1 | 7.7 | 15.0 | 5.2 | 13.1 |
| **Vuln** | 26.1 | 12.4 | 19.5 | 10.9 | 18.6 |
| **H-Vuln** | 26.1 | 11.2 | 18.5 | 9.2 | 17.4 |
| ***Albany*** | | | | | |
| **Random** | 11.6 | 3.2 | 6.9 | 3.1 | 6.7 |
| **Trans** | 11.6 | 1.9 | 4.5 | 1.7 | 4.5 |
| **Vuln** | 11.6 | 4.9 | 8.4 | 4.9 | 8.6 |
| **H-Vuln** | 11.6 | 4.9 | 8.5 | 4.8 | 8.8 |

**Table S24 - Hospitalisation per 100,000 population in Agincourt and Albany for alternative Pre-existing immunity assumption**

| **Priority Strategy** | **No vaccination** | **TIV** | **QIV** |
| --- | --- | --- | --- |
| ***Agincourt*** | | | |
| **Random** | 61.9 | 22.9 | 17.5 |
| **Trans** | 61.9 | 16.8 | 11.2 |
| **Vuln** | 61.9 | 31.4 | 27.2 |
| **H-Vuln** | 61.9 | 27.5 | 22.5 |
| ***Albany*** | | | |
| **Random** | 202.9 | 64.7 | 60.7 |
| **Trans** | 202.9 | 31.4 | 27.7 |
| **Vuln** | 202.9 | 94.6 | 91.7 |
| **H-Vuln** | 202.9 | 94.2 | 91.2 |

**Table S25 - Deaths per 100,000 population in Agincourt and Albany** **for alternative Pre-existing immunity assumption**

| **Priority Strategy** | **No vaccination** | **TIV** | **QIV** |
| --- | --- | --- | --- |
| ***Agincourt*** | | | |
| **Random** | 23.7 | 8.7 | 6.6 |
| **Trans** | 23.7 | 6.1 | 4.0 |
| **Vuln** | 23.7 | 11.9 | 10.3 |
| **H-Vuln** | 23.7 | 10.7 | 8.9 |
| ***Albany*** | | | |
| **Random** | 10.6 | 3.4 | 3.2 |
| **Trans** | 10.6 | 1.7 | 1.5 |
| **Vuln** | 10.6 | 4.7 | 4.6 |
| **H-Vuln** | 10.6 | 4.7 | 4.6 |

# Additional Discussion

## Herd immunity effects

Without indirect effects, untargeted random vaccination (vaccine having 65% efficacy and 15% coverage) would reduce an unmitigated 5% attack rate to approximately 4.5%. For example, if 1000 people became ill in the no-vaccination scenario, 150 of those would have been vaccinated at 15% coverage, and a 65% efficacy vaccine would have protected 98 of the 150.

Due to the herd immunity effect we find that vaccination of a random 15% of the population reduces the attack rate to 1.5%, while a vaccination strategy that targets the most vulnerable age groups reduces the attack rate to 2.6%; this lower effectiveness is because the vulnerable-first strategy preferentially vaccinates those who are less responsible for transmission, that is, not the school age children who contribute most to transmission. Although this indirect effect seems large, it is not an artefact of the particular epidemic model used in this study.

For example, the studies of Weycker et al., Medlock et al., Baguelin et al., Newall et al. and Eichner et al which also used dynamic influenza transmission models found indirect effects of similar magnitude ^51-55^. The most comparable model is that of Eichner et al. Like the Albany model used in our study, the incidence of influenza was calibrated taking into account TIV usage in Germany, which is similar to that of Australia. From the results reported it can be inferred that in the German model the annual symptomatic attack rate would be ~5.5% without TIV, and that the combination of pre-existing immunity (27%) and TIV reduced the attack rate to 2.6%. A similar scenario using our Australian model gives similar results: an attack rate of 5% in the absence of TIV, and find that the combination of TIV and pre-existing immunity reduce the attack rate to 2.1%.

Analysis of historical data on mass vaccination of school children in Japan showed substantial herd immunity benefits. A 2006 survey of published studies found that vaccination of children could produce significant health benefits in the community ^56^; several more recent studies trials support this conclusion ^57, 58^.

## Hospitalisation and Mortality Rates

Results from the Australian model show a higher influenza-related hospitalisation rate than in South Africa. We use Australian age-specific influenza/pneumonia population hospitalisation data to determine the hospitalisation rate. For all ages combined this rate is 42.1 per 100,000; when age-specific hospitalisation rates are applied to the Albany model population, the rate is 55.8 per 100,000, which is slightly higher due to the Albany population age distribution being somewhat older that the Australian average. Note that this population hospitalisation rate (55.8 per 100,000) is not a prediction of the model, rather the per-case hospitalisation rate was selected to achieve this value, where the number of cases was generated from a scenario where TIV usage matched that of Australia (i.e. 80% coverage in the 65+ age group and 10% in other ages groups).

When we extrapolate to a no vaccination scenario we find a hospitalisation rate of 215 per 100,000, three times higher than for South African. Examination of the age breakdown of hospitalisations (Supporting Information Tables S8 and S9) shows that the numbers hospitalised under age 45 is 51.6 and 46.1 per 100,000 population in South Africa and Australia respectively.

This indicates that the higher Australian hospitalisation rate due is to hospitalisation of more older adults, which is caused by two factors. Firstly, the Australian population is comprised of a higher proportion of older individuals compared to South Africa: the proportion of the population over the age of 65 in Australia and South Africa is 13.7% and 5.7% respectively. Secondly Australia has a greater facility to hospitalise older people experiencing complications due to influenza. The low case/hospitalisation ratio for the elderly in South Africa used in this study was derived from the public sector and is appropriate for the rural, low-income community modelled ^8^. Data applicable to the private hospital system found substantially higher hospitalisation rates for the elderly, indicating a lower level of care-seeking in the public versus the private sector ^59^.

In the Albany, Australia community the estimated reduction in influenza-associated excess mortality due to current usage of TIV is 8.6 per 100,000 (from 11.6 to 3.0). If 20% TIV vaccination coverage could be achieved in the Agincourt, South Africa community with a policy prioritising vaccination to those with co-morbid conditions and the elderly, influenza associated excess mortality may be reduced by 17.6 per 100,000 (from 26.1 to 8.5). The greater potential to reduce mortality in South Africa compared to Australia can be attributed to the higher prevalence of HIV, which confers a higher risk of hospitalisation and death following influenza infection ^8^. It is also due to the greater prevalence in South Africa of other underlying health conditions (such as tuberculosis and diabetes) which are not modelled explicitly but which are captured in the model through higher higher case fatality rates in the non-HIV infected population.

## HIV/vulnerable first strategy

The HIV/vulnerable-first strategy also results in larger reductions in attack rate, hospitalisation and death in South Africa compared to Australia at low coverage levels of 2% or 5%. For example with 2% coverage the attack rate is reduced by 14% (from 4.9% to 4.2%) in South Africa, but by only 4% (from 4.9% to 4.7%) in Australia. In South Africa, the highest priority of this strategy is HIV positive individuals, which results in the vaccine being distributed primarily to young adults. In Australia where HIV prevalence is lower, the same strategy distributes vaccines to the elderly and the very young. As young adults are responsible for more influenza transmission than the very young and elderly, this low vaccination strategy is more effective in reducing the symptomatic attack rate, hospitalisation and death in South Africa compared to Australia. At higher coverage levels (15% and 20%) vaccine distribution covers a wider range of ages in both communities, and attack rate reductions are more similar. For example with 20% coverage the attack rate is reduced by 61% in South Africa and by 59% in Australia.

# References

(1) Milne GJ, Kelso JK, Kelly HA, Huband ST, McVernon J. A small community model for the transmission of infectious diseases: comparison of school closure as an intervention in individual-based models of an influenza pandemic. PLoS One. 2008;3(12):e4005.

(2) Milne G, Baskaran P, Halder N, Karl S, Kelso J. Pandemic influenza in Papua New Guinea: a modelling study comparison with pandemic spread in a developed country. BMJ Open. 2013;3:e002518.

(3) Tempia S, Walaza S, Viboud C, Cohen AL, Madhi SA, Venter M, et al. Mortality Associated With Seasonal and Pandemic Influenza and Respiratory Syncytial Virus Among children< 5 Years of Age in a High HIV Prevalence Setting—South Africa, 1998–2009. Clin Infect Dis. 2014;58(9):1241-9.

(4) Cohen C, Simonsen L, Kang J-W, Miller M, McAnerney J, Blumberg L, et al. Elevated influenza-related excess mortality in South African elderly individuals, 1998–2005. Clin Infect Dis. 2010;51(12):1362-9.

(5) Kahn K, Collinson MA, Gómez-Olivé FX, Mokoena O, Twine R, Mee P, et al. Profile: Agincourt health and socio-demographic surveillance system. Int J Epidemiol. 2012;41(4):988-1001.

(6) Kahn K, Tollman SM, Collinson MA, Clark SJ, Twine R, Clark BD, et al. Research into health, population and social transitions in rural South Africa: Data and methods of the Agincourt Health and Demographic Surveillance System1. Scand J Public Health. 2007;35(69 suppl):8-20.

(7) Statistics South Africa. Statistics South Africa <http://www.statssa.gov.za> [cited 2013 2nd December]. Available from: <http://www.statssa.gov.za>.

(8) Cohen C, Moyes J, Tempia S, Groom M, Walaza S, Pretorius M, et al. Severe influenza-associated respiratory infection in high HIV prevalence setting, South Africa, 2009-2011. Emerg Infect Dis. 2013;19(11):1766-74.

(9) Halder N, Kelso JK, Milne GJ. Developing guidelines for school closure interventions to be used during a future influenza pandemic. BMC Infect Dis. 2010;10:221.

(10) Milne G, Kelso J, Kelly H. Strategies for mitigating an influenza pandemic with pre-pandemic H5N1 vaccines. Journal of the Royal Society Interface. 2010;7(45):573-86.

(11) Milne GJ, Halder N, Kelso JK. The cost effectiveness of pandemic influenza interventions: a pandemic severity based analysis. PLoS One. 2013;8(4):e61504.

(12) Kelso J, Halder N, Milne G. Vaccination strategies for future influenza pandemics: a severity-based cost effectiveness analysis. BMC Infect Dis. 2013;18:81.

(13) Halder N, Kelso JK, Milne GJ. A model-based economic analysis of pre-pandemic influenza vaccination cost-effectiveness. BMC Infect Dis. 2014;14(1):266.

(14) The Kirby Institute. HIV, viral hepatitis and sexually transmissible infections in Australia Annual Surveillance Report 2014. Sydney, NSW, Australia: The Kirby Institute, UNSW, 2014.

(15) AVERT. Australia HIV & AIDS Statistics 2014 [cited 2014 7th November]. Available from: <http://www.avert.org/australia-hiv-aids-statistics.htm>.

(16) Hayward AC, Fragaszy EB, Bermingham A, Wang L, Copas A, Edmunds WJ, et al. Comparative community burden and severity of seasonal and pandemic influenza: results of the Flu Watch cohort study. The Lancet Respiratory Medicine. 2014;2(6):445-54.

(17) Horby P, Fox A, Thai PQ, Yen NTT, Hang NLK, Duong TN, et al. The Epidemiology of Interpandemic and Pandemic Influenza in Vietnam, 2007–2010 The Ha Nam Household Cohort Study I. Am J Epidemiol. 2012;175(10):1062-74.

(18) Carrat F, Vergu E, Ferguson NM, Lemaitre M, Cauchemez S, Leach S, et al. Time lines of infection and disease in human influenza: a review of volunteer challenge studies. Am J Epidemiol. 2008;167(7):775-85.

(19) Cauchemez S, Valleron A-J, Boelle P-Y, Flahault A, Ferguson NM. Estimating the impact of school closure on influenza transmission from Sentinel data. Nature. 2008;452(7188):750-4.

(20) Longini IM, Koopman JS, Haber M, Cotsonis GA. Statistical inference for infectious diseases risk-specific household and community transmission parameters. Am J Epidemiol. 1988;128(4):845-59.

(21) Simpson CR, Ritchie LD, Robertson C, Sheikh A, McMenamin J. Effectiveness of H1N1 vaccine for the prevention of pandemic influenza in Scotland, UK: a retrospective observational cohort study. Lancet Infect Dis. 2012;12(9):696-702.

(22) Widgren K, Magnusson M, Hagstam P, Widerström M, Örtqvist Å, Einemo I, et al. Prevailing effectiveness of the 2009 influenza A (H1N1) pdm09 vaccine during the 2010/11 season in Sweden. Euro Surveill. 2013;18(15):20447.

(23) Breteler JK, Tam JS, Jit M, Ket JC, De Boer MR. Efficacy and effectiveness of seasonal and pandemic A (H1N1) 2009 influenza vaccines in low and middle income countries: A systematic review and meta-analysis. Vaccine. 2013;31(45):5168-77.

(24) Actuarial Society of South Africa (ASSA). AIDS and Demographic model 2008 [cited 2015 4th August]. Available from: <http://www.actuarialsociety.org.za/Societyactivities/CommitteeActivities/AidsCommittee/Models.aspx>.

(25) Newall AT, Scuffham PA. Influenza-related disease: the cost to the Australian healthcare system. Vaccine. 2008;26(52):6818-23.

(26) Newall AT, Wood JG, MacIntyre CR. Influenza-related hospitalisation and death in Australians aged 50 years and older. Vaccine. 2008;26(17):2135-41.

(27) Muscatello DJ, Newall AT, Dwyer DE, MacIntyre CR. Mortality attributable to seasonal and pandemic influenza, Australia, 2003 to 2009, using a novel time series smoothing approach. PLoS One. 2013;8(6):e64734.

(28) McAnerney JM, Cohen C, Moyes J, et al. Twenty-five Years of Outpatient Influenza Surveillance in South Africa, 1984-2008. J Infect Dis. 2012;206(suppl 1):S153-S8.

(29) Barr IG, Jelley LL. The Coming Era of Quadrivalent Human Influenza Vaccines. Drugs. 2012;72(17):2177-85.

(30) The WHO Collaborating Centre for Reference and Research on Influenza. The WHO Collaborating Centre for Reference and Research on Influenza (VIDRL) 2012 [cited 2012 18th October]. Available from: <http://www.influenzacentre.org/>.

(31) Australian Institute of Health and Welfare. 2009 Adult Vaccination Survey: summary of results. Canberra: Australian Institute of Health and Welfare, 2011 ISBN 978-1-74249-124-0.

(32) Quinn E, Jit M, Newall AT. Key issues and challenges in estimating the impact and cost-effectiveness of quadrivalent influenza vaccination. Expert Rev Pharmacoecon Outcomes Res. 2014(0):1-11.

(33) WHO. WHO recommendations on the composition of influenza virus vaccines 2014 [cited 2014 7th November]. Available from: <http://www.who.int/influenza/vaccines/virus/recommendations/en/>.

(34) Osterholm MT, Kelley NS, Sommer A, Belongia EA. Efficacy and effectiveness of influenza vaccines: a systematic review and meta-analysis. The Lancet infectious diseases. 2012;12(1):36-44.

(35) Sullivan SG, Feng S, Cowling BJ. Potential of the test-negative design for measuring influenza vaccine effectiveness: a systematic review. Expert review of vaccines. 2014;13(12):1571-91.

(36) McLean HQ, Thompson MG, Sundaram ME, Kieke BA, Gaglani M, Murthy K, et al. Influenza vaccine effectiveness in the United States during 2012–2013: variable protection by age and virus type. J Infect Dis. 2014(211):1529-40.

(37) Kelly HA, Sullivan SG, Grant KA, Fielding JE. Moderate influenza vaccine effectiveness with variable effectiveness by match between circulating and vaccine strains in Australian adults aged 20–64 years, 2007–2011. Influenza Other Respi Viruses. 2013;7(5):729-37.

(38) Skowronski DM, Janjua NZ, Sabaiduc S, De Serres G, Winter A-L, Gubbay JB, et al. Influenza A/subtype and B/lineage effectiveness estimates for the 2011-12 trivalent vaccine: cross-season and cross-lineage protection with unchanged vaccine. J Infect Dis. 2014(210):126-37.

(39) Kissling E, Valenciano M, Larrauri A, Oroszi B, Cohen JM, Nunes B, et al. Low and decreasing vaccine effectiveness against influenza A(H3) in 2011/2012 among vaccination target groups in Europe: results from the I-MOVE multicentre case-control study. Euro Surveill. 2013;18(5):pii=20390.

(40) Pebody RG, Andrews N, McMenamin J, Durnall H, Ellis J, Thompson CI, et al. Vaccine Effectiveness on 2011 2012 trivalent seasonal influenza vaccine in preventing laboratory-confirmed influenza in primary care in the United Kingdom: evidence of waning intra-seasonal protection. Euro Surveill. 2013;18(5):pii=20389.

(41) Castilla J, Martínez-Baz I, Martínez-Artola V, Reina G, Pozo F, García Cenoz M, et al. Decline in influenza vaccine effectiveness with time after vaccination. Euro Surveill. 2013;18(5):pii=20388.

(42) WHO. Influenza Vaccine Use 2014 [cited 2014 November 13th]. Available from: <http://www.who.int/influenza/vaccines/use/en/>.

(43) Ritzwoller DP, Bridges CB, Shetterly S, Yamasaki K, Kolczak M, France EK. Effectiveness of the 2003–2004 influenza vaccine among children 6 months to 8 years of age, with 1 vs 2 doses. Pediatrics. 2005;116(1):153-9.

(44) DiazGranados CA, Denis M, Plotkin S. Seasonal influenza vaccine efficacy and its determinants in children and non-elderly adults: a systematic review with meta-analyses of controlled trials. Vaccine. 2012;31(1):49-57.

(45) Su S, Chaves SS, Perez A, D'Mello T, Kirley PD, Yousey-Hindes K, et al. Comparing clnical characteristics between hospitalized adults with laboratory-confirmed influenza A and B virus infection. Clin Infect Dis. 2014;59(2):252-5.

(46) Simmerman JM, Chittaganpitch M, Levy J, Chantra S, Maloney S, Uyeki T, et al. Incidence, seasonality and mortality associated with influenza pneumonia in Thailand: 2005–2008. PLoS One. 2009;4(11):e7776.

(47) Cohen C, Moyes J, Tempia S, Groome M, Walaza S, Pretorius M, et al. Mortality amongst Patients with Influenza-Associated Severe Acute Respiratory Illness, South Africa, 2009-2013. PLoS One. 2015;10(3):e0118884.

(48) Hall CE, Cooney MK, Fox JP. The Seattle virus watch iv. comparative epidemiologic observations of infections with influenza A and B viruses, 1965–1969, in families with young children. Am J Epidemiol. 1973;98(5):365-80.

(49) Frank AL, Taber LH. Variation in frequency of natural reinfection with influenza A viruses. J Med Virol. 1983;12(1):17-23.

(50) Frank AL, Taber LH, Porter CM. Influenza B virus reinfection. Am J Epidemiol. 1987;125(4):576-86.

(51) Weycker D, Edelsberg J, Halloran ME, Longini IM, Nizam A, Ciuryla V, et al. Population-wide benefits of routine vaccination of children against influenza. Vaccine. 2005;23(10):1284-93.

(52) Medlock J, Galvani AP. Optimizing influenza vaccine distribution. Science. 2009;325(5948):1705-8.

(53) Baguelin M, Flasche S, Camacho A, Demiris N, Miller E, Edmunds WJ. Assessing optimal target populations for influenza vaccination programmes: an evidence synthesis and modelling study. PLoS Med. 2013;10(10):e1001527.

(54) Newall AT, Dehollain JP, Creighton P, Beutels P, Wood JG. Understanding the cost-effectiveness of influenza vaccination in children: methodological choices and seasonal variability. Pharmacoeconomics. 2013;31(8):693-702.

(55) Eichner M, Schwehm M, Hain J, Uphoff H, Salzberger B, Knuf M, et al. 4Flu-an individual based simulation tool to study the effects of quadrivalent vaccination on seasonal influenza in Germany. BMC Infect Dis. 2014;14(1):365.

(56) Jordan R, Connock M, Albon E, Fry-Smith A, Olowokure B, Hawker J, et al. Universal vaccination of children against influenza: are there indirect benefits to the community?: a systematic review of the evidence. Vaccine. 2006;24(8):1047-62.

(57) King Jr JC, Stoddard JJ, Gaglani MJ, Moore KA, Magder L, McClure E, et al. Effectiveness of school-based influenza vaccination. N Engl J Med. 2006;355(24):2523-32.

(58) Loeb M, Russell ML, Moss L, Fonseca K, Fox J, Earn DJ, et al. Effect of influenza vaccination of children on infection rates in Hutterite communities: a randomized trial. JAMA. 2010;303(10):943-50.

(59) Kyeyagalire R, Tempia S, Cohen AL, Smith AD, McAnerney JM, Dermaux-Msimang V, et al. Hospitalizations associated with influenza and respiratory syncytial virus among patients attending a network of private hospitals in South Africa, 2007–2012. BMC Infect Dis. 2014;14(1):694.
